# Maize Inbreds Exhibit High Levels of Copy Number Variation (CNV) and Presence/Absence Variation (PAV) in Genome Content

**DOI:** 10.1371/journal.pgen.1000734

**Published:** 2009-11-20

**Authors:** Nathan M. Springer, Kai Ying, Yan Fu, Tieming Ji, Cheng-Ting Yeh, Yi Jia, Wei Wu, Todd Richmond, Jacob Kitzman, Heidi Rosenbaum, A. Leonardo Iniguez, W. Brad Barbazuk, Jeffrey A. Jeddeloh, Dan Nettleton, Patrick S. Schnable

**Affiliations:** 1 Department of Plant Biology, University of Minnesota, Saint Paul, Minnesota, United States of America; 2 Interdepartmental Genetics Graduate Program, Iowa State University, Ames, Iowa, United States of America; 3 Department of Genetics, Development, and Cell Biology, Iowa State University, Ames, Iowa, United States of America; 4 Department of Agronomy, Iowa State University, Ames, Iowa, United States of America; 5 Center for Carbon Capturing Crops, Iowa State University, Ames, Iowa, United States of America; 6 Department of Statistics, Iowa State University, Ames, Iowa, United States of America; 7 Center for Plant Genomics, Iowa State University, Ames, Iowa, United States of America; 8 Interdepartment Plant Biology, Iowa State University, Ames, Iowa, United States of America; 9 Roche NimbleGen, Madison, Wisconsin, United States of America; 10 University of Florida, Gainesville, Florida, United States of America; The Salk Institute for Biological Studies, United States of America

## Abstract

Following the domestication of maize over the past ∼10,000 years, breeders have exploited the extensive genetic diversity of this species to mold its phenotype to meet human needs. The extent of structural variation, including copy number variation (CNV) and presence/absence variation (PAV), which are thought to contribute to the extraordinary phenotypic diversity and plasticity of this important crop, have not been elucidated. Whole-genome, array-based, comparative genomic hybridization (CGH) revealed a level of structural diversity between the inbred lines B73 and Mo17 that is unprecedented among higher eukaryotes. A detailed analysis of altered segments of DNA conservatively estimates that there are several hundred CNV sequences among the two genotypes, as well as several thousand PAV sequences that are present in B73 but not Mo17. Haplotype-specific PAVs contain hundreds of single-copy, expressed genes that may contribute to heterosis and to the extraordinary phenotypic diversity of this important crop.

## Author Summary

There is a growing appreciation for the role of genome structural variation in creating phenotypic variation within a species. Comparative genomic hybridization was used to compare the genome structures of two maize inbred lines, B73 and Mo17. The data reinforce the view that maize is a highly polymorphic species, but also show that there are often large genomic regions that have little or no variation. We identify several hundred sequences that, while present in both B73 and Mo17, have copy number differences in the two genomes. In addition, there are several thousand sequences, including at least 180 sequences annotated as single-copy genes, that are present in one genome but entirely missing in the other genome. This genome content variation leads to differences in transcript content between inbred lines and likely contributes to phenotypic diversity and heterosis in maize.

## Introduction

Although many analyses of genetic variation have focused on single nucleotide polymorphisms (SNPs), there is a growing appreciation for the roles of structural variation as a cause for phenotypic variation [1]–[7]. Indeed, structural variation can have major phenotypic consequences [6]. The term copy number variation has been used to describe duplications, deletions and insertions among individuals of a species [5]. Herein the term copy number variation (CNV) is reserved to describe sequences that are present in both genomes being compared, albeit in different copy number. The term presence-absence variation (PAV) is used to describe sequences that are present in one genome but entirely missing in the other genome.

Maize is phenotypically diverse [8]–[9] and this phenotypic diversity is reflected by substantial variation in phenotypic and transcript levels among maize lines [8], [10]–[11]. In addition, the maize genome exhibits extraordinarily high levels of genetic diversity as assayed at the level of SNPs, InDel Polymorphisms (IDPs), and structural variation [9],[12]. The frequency of SNPs among maize inbreds is higher than the frequency of SNPs between humans and chimpanzees [9]. The inbred lines B73 and Mo17 are important models for the structural and functional genomics of maize. On average, B73 and Mo17 contain an IDP every ∼300 bp and SNPs every ∼80 bp [13]–[14] and within transcripts SNPs are found between the inbred lines B73 and Mo17 on average every 300 bp [15]. These levels of diversity are not limited to comparisons between B73 and Mo17. When comparing any two randomly chosen maize inbred lines, there is, on average, one polymorphism every 100 bp [16]–[17]. Collectively, these studies indicate that maize has relatively high levels of SNPs and IDPs as compared to many other species [9].

There is also cytogenetic evidence for structural variation in the genomes of maize inbreds. Structural genomic variation involves alterations in DNA sequence beyond SNPs or small IDPs, and includes large-scale differences in chromosomal structure, altered locations of genes or repetitive elements, copy number variation (CNV) and presence/absence differences among haplotypes. Large-scale differences in chromosomal structure between maize inbred lines were first identified through cytogenetic studies. Barbara McClintock and others analyzed heterochromatic knob (highly condensed, tandem repeat regions) content and size to characterize genome variation [18]–[20]. Recent studies have documented differences in the content of several classes of repetitive DNA between maize inbreds at the chromosomal level [21]. Flow cytometry studies have also documented significant variation in overall genome sizes among inbred lines [22].

Sequence-based methodologies have documented structural diversity at a higher resolution (reviewed by [9],[12]). Sequencing of BACs containing the *bz1* gene from eight different inbred lines revealed two significant findings [23]–[24]. First, there is variation for the presence of several genic fragments such that these “genes” are found at this locus in some inbreds but not in others [23]. These “genes” were subsequently found to be gene fragments that had been mobilized by *Helitron* transposons [25]–[26]. These are not PAVs because although a genome may lack a copy in the vicinity of the *bz1* locus, such a genome typically contained one or more copies of these genes (or gene fragments) elsewhere. Second, comparison of multiple haplotypes revealed major differences in the amount and types of repetitive elements between genes. The same gene can be flanked by very different repetitive elements in different inbred lines [23]. At the same time, similar kinds of repeat diversity between haplotypes were reported in the *a1-sh2* interval [27]. Both of these findings have been supported by analyses of other genomic regions in B73 and Mo17 [28]. A study of the presence and location for many genic fragments in B73 and Mo17 BAC libraries suggested that many sequences can vary in location or even presence between B73 and Mo17 [28]. There is also evidence for variation in the presence of nearly identical paralogs (NIPS) in different maize inbred lines [29].

Understanding the intraspecific variation of maize has important implications for crop improvement and plant breeding. Long-term selection experiments have demonstrated a surprising wealth of potential; even when starting with relatively little genetic diversity it has been possible to continue to make phenotypic gains for traits such as oil content for over a century [30]. In addition, the combination of variation from different maize inbred lines in hybrids results in heterosis [31]. The availability of genomic resources for maize, particularly the B73 maize genome sequence [32] has provided an opportunity to conduct genome-wide analyses of structural variation. We have used high-density oligonucleotide microarrays to identify patterns of structural variation across the maize genome. We find evidence for a high rate of CNVs. In addition, we identify several thousand DNA segments, often including genic sequences, that are present in the B73 genome but absent from the Mo17 genome (i.e., PAVs). By assessing genome-wide structural variation in maize we have gained a better understanding of the nature of variation among different maize inbred lines.

## Results

### Development and annotation of a CGH microarray for maize

Genomic variation within a species can be assessed using comparative genomic hybridization (CGH). A high-density (2.1 million feature) oligonucleotide microarray was designed using the sequences of B73 BACs. Probes range in sizes from 45–85 bp were selected using slightly relaxed criteria (due to the overlap of adjacent BAC sequences and lack of assembly at the time of design) relative to those traditionally used for CGH probe design. The 2.12M probes were aligned to the B73 RefGen_v1 [32] released by the maize genome sequencing project (MGSP). It was possible to identify perfect matches (100% ID and 100% coverage) for 93% (1.98M/2.10M) of the probes. Approximately ∼1.78 million of the probes had only a single perfect match and were therefore deemed to be single copy, ∼120k probes had two perfect matches and ∼34k probes had three perfect matches (Figure S1).

**Figure 1:**
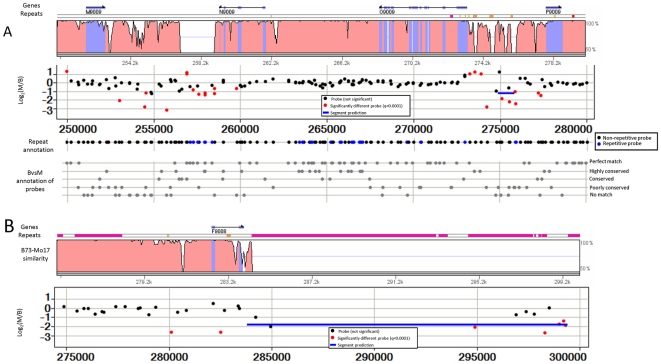
Significant hybridization differences are due to structural variation. (A) The B73 and Mo17 sequences for a portion of the 9,009 locus (sequenced by [28]) were aligned using Vista [71] which displays the percent identity as a sliding window of 100 bp (y-axis is 50% to 100% identity). The location of genes annotated by Brunner et al. [28] (indicated by light blue sequences in the alignment) and repeat elements (the color-coded track right above the alignments; pink indicates retrotransposons and orange indicates transposons) are shown above the VISTA alignment. The log_2_(Mo17 signal/B73 signal) is shown for each probe in this region. The red probes exhibit significantly different (q<0.0001) signal in B73 and Mo17. The blue line indicates a segment with altered hybridization that was identified using DNAcopy. There are also data tracks that display the repeat annotation and B73/Mo17 similarity for each probe. Note that these annotations are based on the genome-wide analysis, not detailed analyses of these regions. In (B) we present the annotation, alignment and CGH data for a portion of the 9008 loci (sequence and annotated by Brunner et al., [28]).

All of these perfectly matched probes were classified based on their repetitiveness and locations relative to predicted genes (see Methods for details and Table 1 for numbers). Approximately 30% of the probes exhibited evidence of containing repetitive sequences (Methods; Table 1). Probes were also mapped relative to genes and other types of annotation produced by the MGSP. The distributions of probes relative to these types of annotation were assessed by visualizing the locations of probes that aligned to several genomic regions for which high-quality assembled sequence and manual annotation were available for both B73 and Mo17 (Figure 1 and Figure S2; [23],[28]). There are 1,604 probes within the ∼1 Mb of B73 sequence from these four regions (selected portions are shown in Figure 1 and Figure S2). Probe density is generally high for those regions in which the B73 and Mo17 haplotypes align well. These regions tend to be genic or low copy and have 3–4 probes per kb. Consistent with the NimbleGen probe design strategy, fewer probes are located in regions consisting of a high percentage of repetitive element sequences.

**Table 1:** Probe classifications and enrichment in specific categories.

Classifications	Categories	# of Probes	Significant probes (q<0.0001)	B>M significant probes	M>B significant probes
All probes		2,110,668	325,813	291,963	33,850
Probe classification by “repetitiveness”^a^	Non-repeat	1,461,771 (69%)	278,390 (85%)	249,188 (85%)	29,202 (86%)
	Total repetitive	630,586 (30%)	47,423 (15%)	42,775 (15%)	4,648 (14%)
	Cereal repeat	226,706 (11%)	12,349 (4%)	11,586 (4%)	763 (2%)
	Crosshyb	585,105 (28%)	40,907 (13%)	36,616 (13%)	4,291 (13%)
	Multi-copy	54,791 (3%)	6,276 (2%)	5,497 (2%)	779 (2%)
Probe classification by Mo17 conservation annotation^b^	Perfect match (100%)	871,664 (41%)	44,062 (14%)	22,586 (8%)	21,476 (63%)
	Highly Conserved (>97%)	331,590 (16%)	42,992 (13%)	36,659 (13%)	6,333 (19%)
	Conserved (>90%)	505,519 (24%)	106,996 (33%)	103,817 (35%)	3,179 (9%)
	Poorly conserved (>75%)	182,570 (9%)	55,410 (17%)	53,929 (18%)	1,481 (4%)
	No match in Mo17 (<75%)	219,325 (10%)	76,353 (23%)	74,972 (26%)	1,381 (4%)
Genic annotation	Exon	98,886 (4.5%)	11,885 (3.6%)	9,803 (3.4%)	2,082 (6.2%)
	Exon-intron	62,448 (2.8%)	8,447 (2.6%)	7,134 (2.4%)	1,313 (3.9%)
	Intron	145,483 (6.6%)	24,047 (7.4%)	21,159 (7.2%)	2,888 (8.5%)
	3′ 2000bp	121,133 (5.5%)	26,682 (8.2%)	24,569 (8.4%)	2,113 (6.2%)
	5′ 2000bp	134,408 (6.1%)	29,284 (9%)	26,618 (9.1%)	2,666 (7.9%)
	Intergene	1,650,875 (74.6%)	225,468 (69.2%)	202,680 (69.4%)	22,788 (67.3%)

a The repetitive nature of each probe was determined by comparing to the B73 reference genome. All probes that satisfy criteria (see Methods for details) for cereal repeat, crosshyb or multi-copy were designated as repetitive.

b The probes were each classified based upon the most significant similarity to the Mo17 WGS sequence from JGI. The full definition for each category can be found in the Methods section.

Analyses of these regions were used to evaluate the quality of our genome-wide probe annotation. Several tracks in Figure 1A provide information about the repetitive and genic classifications of probes. Our genome-wide annotations generally agree with the detailed annotation information available for these four regions. The probes that were classified as repetitive in the genome-wide analyses were often found within sequences that were annotated as repetitive or retrotransposon based on the four regions that had been subjected to manual annotation.

All probes were designed based on the B73 haplotype. To determine whether probe sequences were conserved in Mo17, probe sequences were aligned to a collection of 42,206,644 Mo17 whole-genome shotgun (WGS) reads generated by the DOE's Joint Genome Institute (JGI) and provided to us prior to publication by the Rohksar group. Based on these alignments each probe was classified as being a perfect match (100% identity and coverage), highly conserved (>97% identity and coverage), conserved (>90% identity and coverage), poorly conserved (>75% identity and >70% coverage) or as having no significant match in the JGI Mo17 data set. Over 80% of the probes were at least 90% identical to Mo17 sequences with over 90% of probe sequence coverage (Table 1).

The analysis of the four regions that have complete coverage of the Mo17 haplotype permitted us to compare the results of our genome-wide classifications with actual alignments of complete B73 and Mo17 sequences. Because the JGI collection of Mo17 WGS reads provides approximately 4× coverage of the genome we expect some probes to be mis-classified as poorly conserved or as having no match in Mo17 simply due to incomplete sampling of the Mo17 genome. Overall, there was strong agreement between our classification of probes based on alignments to the Mo17 WGS reads and the genomic alignments shown in Figure 1. As expected, there were few cases of probes within highly conserved regions that had erroneously been classified as poorly conserved or no match. Even so, most probes within regions of the B73 haplotype for which there was no significant similarity in allelic regions of the Mo17 haplotype did not match the WGS Mo17 sequences. Some of the probes that matched regions of the B73 haplotype for which there was no significant similarity in allelic regions of the Mo17 haplotype (i.e., positions 257,000–259,000 in Figure 1A) did have similarity to WGS Mo17 sequences. This suggests that regions of the B73 haplotype that can not be aligned to allelic positions of the Mo17 haplotype are of two types. In some cases the non-aligning sequences are B73-specific (PAVs), while in other cases Mo17 contains these sequences but at non-allelic positions similar to those reported by Fu and Dooner [23].

### Structural variation detected by CGH

B73 and Mo17 genomic DNA samples were hybridized to the microarray using dye swaps as well as technical replication (Methods). Analysis of the CGH data reveals a bias towards stronger hybridization signals from B73 genomic DNA than from Mo17 genomic DNA (Figures S3, S4). This bias is likely due to the fact that the array design was based upon the B73 genomic sequence and that polymorphisms between B73 probes and the labeled Mo17 genomic DNA may reduce signal strength. This imbalance in signals between the genotypes violates an assumption required to perform typical global normalization. Consequently, we implemented a normalization procedure that utilized a subset of probes for array normalization. This strategy employed the raw signals from those 840,289 probes whose sequences are absolutely conserved between B73 and Mo17 (based on our analysis of the Mo17 WGS data) to normalize the remaining data (∼60% of the probes). A linear model was used to estimate the signal from each genotype and to determine q-values to control false discovery rates.

To understand the biological causes of differences in hybridization signals between B73 and Mo17 we initially focused on the four regions shown in Figure 1 and Figure S2 for which high-quality B73 and Mo17 sequence were available. We found significant (q<0.0001) differences in hybridization signal in B73 relative to Mo17 for 234 of the 1,604 probes within these regions (Table 2). As expected it was much more common to observe higher hybridization signal in B73 (210 probes) than the reverse (24 probes).

**Table 2:** Influence of polymorphisms on hybridization variation.

# SNPs	# Probes	B>M significant probes	M>B significant probes	Average log2(M/B)
0	568	6 (1%)	6	0.000
1	180	13 (7%)	6	−0.196
2	95	18 (19%)	2	−0.400
3	67	17 (25%)	1	−0.552
4	54	14 (26%)	0	−0.733
5 or more	640	142 (22%)	9	−0.789
Total	1604	210 (13%)	24	

*: Significant probes indicate a q value<0.0001 from the linear model.

There are at least three biological reasons why a probe exhibits significant differences in signal after being hybridized to genomic DNA from two inbred lines. First, the probe sequence may have polymorphisms in the two genotypes (SNPs and IDPs). Second, the copy number of the probe in the genomic DNA might be different in the two genotypes being compared (CNV). Third, the probe sequence may be present in the genomic DNA of the reference genotype but not the other (PAV). It is important to remember that while all three reasons could explain why a probe would have a higher signal in B73 than in Mo17, only the second reason is likely to cause probes to have higher signals in Mo17 than in B73 because all probes were designed based on the B73 sequence.

The impact of sequence polymorphisms on hybridization can be observed by comparing the average log_2_(Mo17/B73) in probes with different levels of polymorphism between B73 and Mo17 (Table 2). For probes with no polymorphisms the average log_2_(Mo17/B73) is zero. As the number of polymorphisms between B73 and Mo17 increases, the log_2_(Mo17/B73) value decreases and the percentage of probes that exhibit statistically significant differences in signal strength (q<0.0001) increases. Most of the probes with significant variation (68%) have 5 or more SNPs (note that often these probes can not be aligned to the Mo17 WGS reads at all and many have multiple IDPs or may even be absent altogether from Mo17). Overall, this finding indicates that the majority of the significant differences in hybridization signals are due to the presence of multiple polymorphisms within the ∼70 bp probe sequence or due to sequences that encompass or overlap the probe sequence that are present in B73 but absent from the Mo17 genome.

Further support for the concept that many of the probes that exhibit significant differences in hybridization signals are reporting structural variation was provided by visualization of the distribution of log_2_(Mo17/B73) signals relative to the four B73/Mo17 haplotype alignments (Figure 1 and Figure S2). For example, each of the four probes in Figure S2A that have significantly lower signals in Mo17 than in B73 are in regions in which the two haplotypes differ substantially. Similarly, in Figure S2B the six probes with significantly lower signal in Mo17 than in B73 all fall near regions of structural variation. Many of the probes with significant signal differences between B73 and Mo17 occurred in the regions surrounding non-shared repetitive elements. It was surprising that some of the probes with 5 or more SNPs (in alignments between these two regions only) did not exhibit significant differences in hybridization signals. However, we noted that although some of these probes (several examples shown in Figure S2) do not have a similar sequence at an allelic position in Mo17, they do have one present elsewhere in the Mo17 genome based on alignments to the Mo17 WGS sequences.

### Genome-wide analysis of probes with variable signal in B73 and Mo17

After analyzing in detail probes that aligned to the regions presented in Figure 1 and Figure S2, we assessed the characteristics of all probes that exhibit significant variation in B73 relative to Mo17. At a cut-off of q<0.0001 there are 325,813 probes with significant differences in hybridization signals between B73 and Mo17 (15% of all probes, Table 1). The majority (90%) of these probes exhibit higher signals from B73 than from Mo17 (B>M; Table 1), as can be readily observed in volcano and MA plots (Figure S5). In general, and as expected, repeat probes tend to have higher signals than non-repetitive probes (as seen in MA plots in Figure S6). Both B73>Mo17 and Mo17>B73 probes are enriched for non-repetitive probes and consequently depleted for repetitive probes (Table 1 and Figures S7, S8A). This is not surprising because the signals associated with repetitive probes reflect cross-hybridization from multiple genomic sites and therefore a change at a single site will have less impact on signal strength.

There are striking differences in the B73>Mo17 and Mo17>B73 probes when comparing annotation based on alignments of probe sequences to the Mo17 WGS sequences (Table 1, Figures S8B, S9, S10). Consistent with our analysis of probes from Figure 1, genome-wide B73>Mo17 probes are enriched for sequences with no match or poor conservation in the Mo17 WGS sequence and are correspondingly depleted for highly conserved or identical probes. Those B73>Mo17 probes that do have an identical or highly conserved sequence among the Mo17 WGS sequences are likely to be examples of CNV and can be used to estimate the rate of CNV. The Mo17>B73 probes follow the opposite pattern with enrichment for probes that have a highly conserved or identical sequence in both B73 and Mo17. This indicates that many of the probes with no match in the Mo17 WGS sequence reflect actual sequence differences, not simply a lack of coverage in the Mo17 WGS sequence.

Probes were compared to the full “working set” of genes predicted by the MGSP (www.maizesequence.org). This “permissive” gene set (n = 129,891) includes low-copy transposons as well as pseudogenes. The B73>Mo17 probes exhibit a distribution of genic and intergenic matches that is very similar to all probes. Interestingly, the Mo17>B73 probes are slightly depleted for intergenic probes and show an enrichment for probes near or within genes (Table 1; Figure S8C). A very similar distribution is observed using the filtered set of high-quality gene annotations from the MGSP (data not shown).

### Distribution of structural variation throughout the maize genome

**Figure 2:**
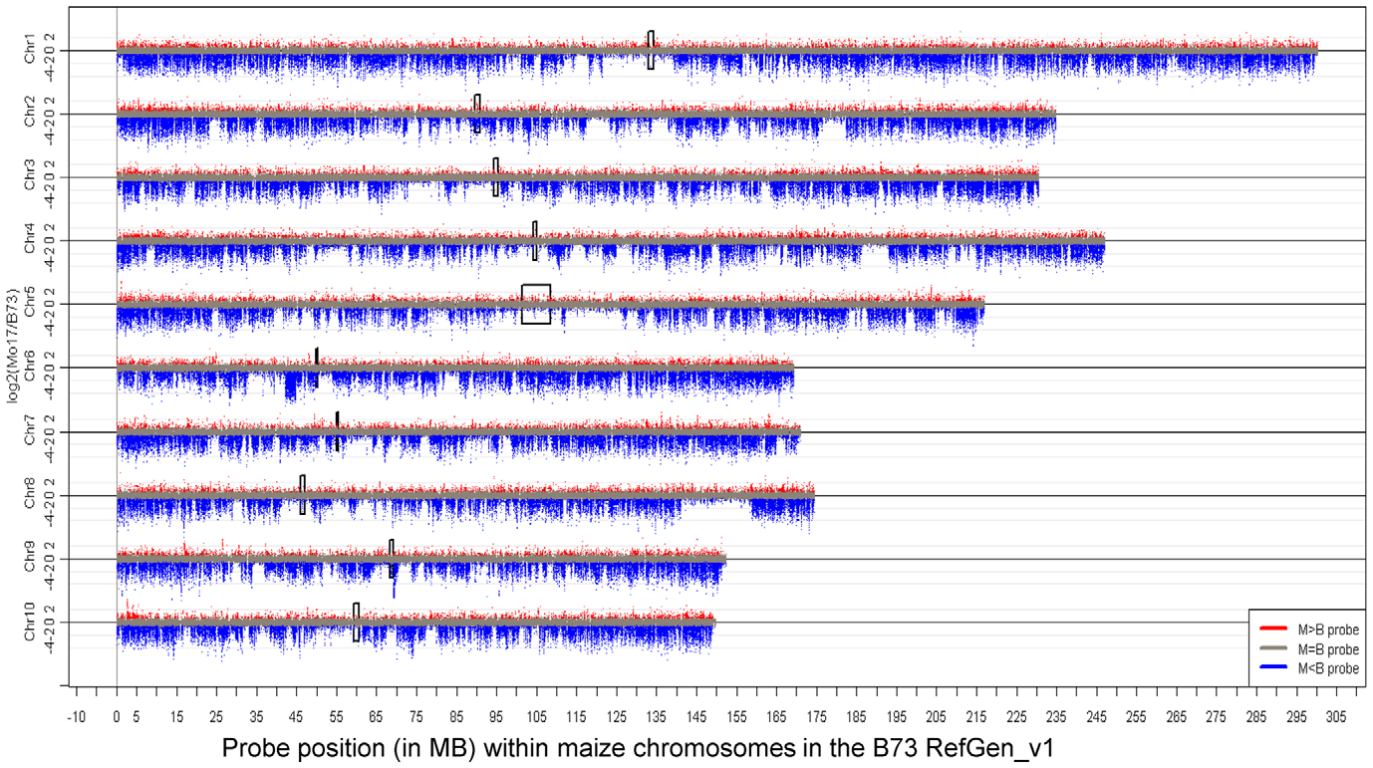
Genomic distribution of log_2_(Mo17/B73) signals. The log_2_(Mo17/B73) hybridization intensities are plotted for each chromosome. Data points below the line indicate higher hybridization in B73 than in Mo17. The positions of the centromeres [72] are indicated by black boxes. Note that there are chromosomal regions with high rates of variation (example near 42–44 MB on chromosome 6) and regions with low rates of variation (example from 140–160 MB on chromosome 8).

**Figure 3:**
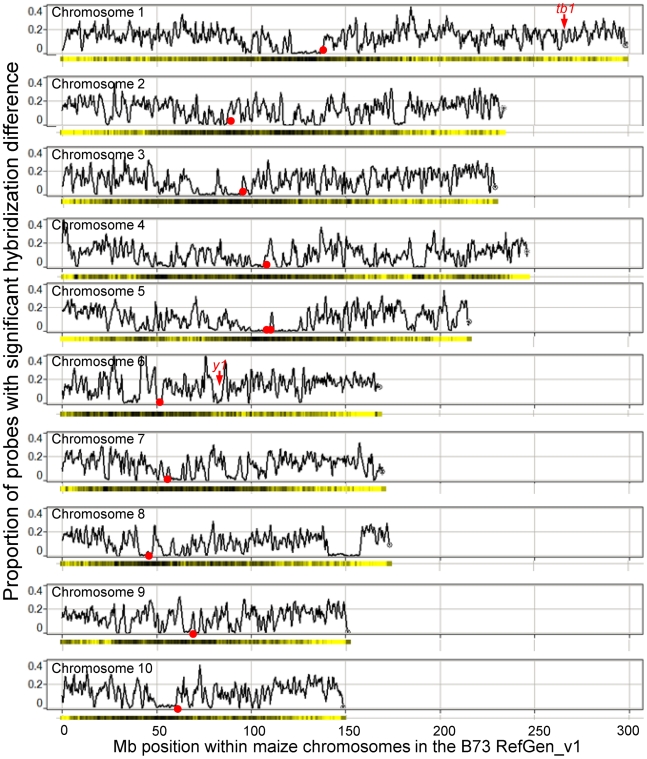
Identification of regions of low structural diversity. The proportion of probes that exhibit significantly higher hybridization to B73 genomic DNA than Mo17 (q<0.0001) was determined for a sliding window of 1 Mb probes with increments of 0.33 Mb. The approximate position of each centromere (from Wolfgruber et al., [72]) is indicated by a red circle on each chromosome. The locations of the *tb1* [41] and *y1* [40] genes, which are known to have undergone selective sweeps, are indicated. The gene density (based on the filtered gene set from the MGSP) is shown below each chromosome. The gene density was determined based on the number of genes per Mb. The dark color indicates low gene density while the yellow color indicates higher gene density.

The probes were aligned to the B73 RefGen_v1 to visualize the patterns of structural variation along the B73 and Mo17 chromosomes (Figure 2). It should be noted that while the B73 reference genome generally place segments of DNA in the proper order at the level of a single BAC, the local orientation and order of sequence contigs within a BAC has not always been determined. Therefore, our genomic localization of the probes is likely only accurate within the average size of a BAC (∼170 kb). The log_2_(Mo17/B73) signals for each probe were plotted relative to the genomic localization of the probes. As noted above, the majority of probes with significant B73>Mo17 hybridization detect structural variation. The genomic view provided in Figure 2 reveals that structural variation between these two inbreds is not evenly distributed throughout the maize genome. The large number of data points plotted on this graph (∼>2 million) can make it difficult to visualize the relative rates of variation across the genome. Therefore, we implemented a sliding window analysis to observe the frequency of probes with significant B73>Mo17 variation in regions that are the approximately the size of 10 average BACs (Figure 3).

There are a number of highly conserved genomic regions that have very little or no structural variation between B73 and Mo17 (Figure 3). For example, there is an ∼19 Mb region on chromosome 8 (Figure S11A; positions 140,904,890–158,897,190) and a 17 Mb region on chromosome 1 (positions 121,420,890–138,984,608) with no evidence for structural variation. The sliding window analysis identified 104 regions that exhibit little to no structural variation (fewer than 4% of the probes exhibit significant variation). Seven of these low diversity regions (on chromosomes 1, 3, 4, 5, 8 and 9) are over 10 Mb.

We performed further characterization of the large regions on chromosomes 1 and 8 with low rates of structural variation. The majority of probes within these regions (83%) are 100% conserved in B73 and Mo17 suggesting that these are low diversity regions. None of 388 primer pairs designed to amplify sequences within these regions revealed sequence variation between B73 and Mo17 that could be detected via agarose gel electrophoresis. In comparison, 13% of all primer sets designed for random genomics sites detect variation. We then used Temperature Gradient Capillary Electrophoresis (TGCE) to test whether B73 and Mo17 amplification products from 156 of the 388 primer pairs from the conserved regions contain SNPs or small IDPs. TGCE is sensitive enough to detect a single SNP in amplicons of over 800 bp and 1 bp IDPs in amplicons of ∼500 bp [33], which is the typical size of these amplification products. Of these 123/156 (79%) exhibited no evidence of even a single SNP or IDP between B73 and Mo17, indicating the high level of sequence conservation within these two intervals. In contrast, only 39% of randomly selected sites are not polymorphic using TCGE assays.

There is a tendency for these large low diversity regions to be located near the central portions of the chromosomes and the centromere to be located near one side of a low diversity region for all chromosomes except 9. However, there are many low diversity regions that are not centromeric (for example, the large region on chromosome 8). These low diversity regions are likely to represent regions in which B73 and Mo17 are identical by descent or regions with no structural variation in the maize species. These low diversity regions also exhibit very low levels of differential gene expression. Only three of the 196 genes from the MGSP filtered gene set that are located in the conserved chromosome 1 or chromosome 8 regions and queried by the Affymetrix 17K microarray exhibit evidence for differential expression in seedling, embryo or endosperm tissue from B73 and Mo17 [11]. The few cases of differential expression within sequence-conserved regions may reflect the action of trans-acting factors that are polymorphic between B73 and Mo17.

### Mega-base sized B73-specific sequence

**Figure 4:**
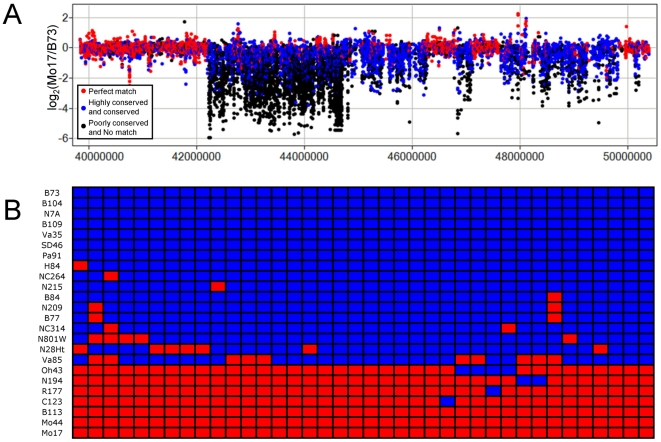
Characterization of 2 Mb region on chromosome 6 that is present in B73 but missing in the Mo17 genome. (A) A 10 Mb region on chromosome 6 is shown. The color-coding for each probe indicates the level of conservation of the probe sequence to the Mo17 WGS sequence. The coordinates on the x-axis refer to base pair position within chromosome 6 of the B73 Refgen_v1. The 2.6 Mb region from 42.2 to 44.8 is enriched for probes that are poorly conserved or have no match in the Mo17 sequence and the majority of these probes exhibit much higher signal in B73 than in Mo17. (B) The data from 38 primer pairs are shown. Blue indicates successful amplification for a particular inbred by primer combination while red indicates no amplification. The full set of 38 primer pairs (see Table S1 for details) amplify products in B73 but not in Mo17.

One visually striking feature in Figure 2 and Figure S11B is the region on chromosome 6 (positions 42,211,131–44,706,565) that contains a cluster of B73>Mo17 probes. Closer inspection of this region indicates that the region of elevated structural variation is ∼2.6 Mb (Figure 4A). The majority of probes in this region are either poorly conserved or not present among the Mo17 454 WGS sequences. This finding suggests that this 2.6 Mb sequence is present in the B73 genome but entirely absent from the Mo17 genome. Primer pairs designed based on the B73 sequence of this region were used to conduct PCR on B73 and Mo17 (Table S1). All 38 primer pairs amplified B73 but not Mo17. These primer pairs were also used to query for the presence of this 2.6 Mb segment in 22 other maize inbred lines. The data suggest that 16 of the inbreds contain this segment while the other 6 did not (Figure 4B). These inbreds seemed to contain (or lack) the entire segment as a haplotype block. It should be noted that both the CGH and PCR analyses suggest that all 2.6 Mb of sequence is missing in its entirety from the Mo17 genome and from the other six inbreds; neither it, nor components of it, are located at non-allelic positions.

Based on the filtered gene set from the MGSP there are 31 genes within the ∼2.6 Mb B73-specific interval. RNA-seq experiments provide evidence for expression of 14/31 genes located within this interval in B73 shoot apical meristem tissue (Yi Jia, Kaz Ohtsu and Patrick S. Schnable, unpublished data) suggesting that many of the genes in this interval may be functional in B73. In addition, three of the genes within this interval are detected by the Affymetrix 17K microarray. The expression of all three of these genes are detected in B73 but not in Mo17 [11]. Notably, intermediate levels of expression of these genes are also detected in B73xMo17 and Mo17xB73 hybrids.

The sequence of the B73-specific region does not exhibit similarity to the chloroplast or mitochondrial genomes. The genes present within this region do not shown synteny to any specific region of the rice genome but are found scattered across different rice chromosomes. Maize chromosome 6 is syntenic to rice chromosomes 5 and 6 [32]. However, there is a region near the centromere that does not show synteny with any rice chromosome and the 2.6 Mb segment is located within this region. Fine-scale analysis of the synteny in this region indicates that the distal sequence shows synteny to rice chromosome 5 while the sequence proximal to the B73-specific sequence is syntenous to rice chromosome 6. Hence, the B73-specific region is right at the point where the syntenic regions of maize chromosome 6 appear to have fused relative to rice chromosomes 5 and 6. The facts that many of these genes are expressed in maize and that many of the genes within this region are conserved in rice implies that the B73-region was likely selected in maize and have been deleted in the Mo17 haplotype.

### Identification of copy number variants and genome content differences

**Figure 5:**
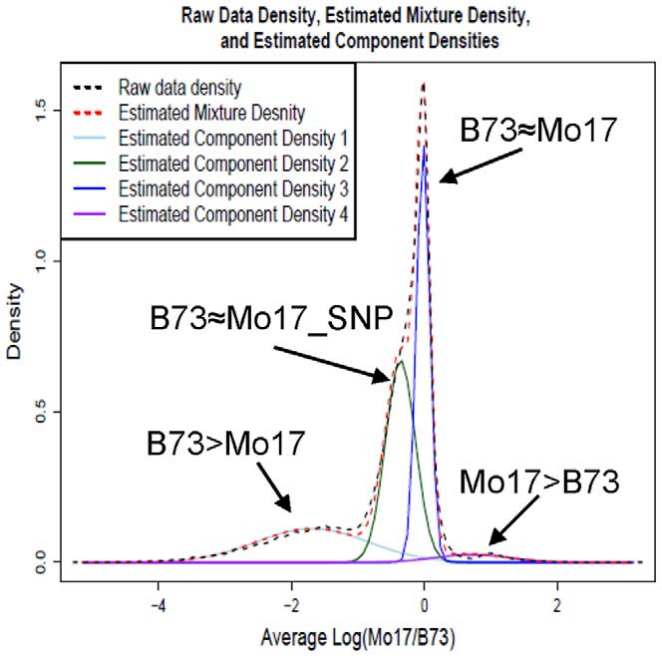
Distribution of average log2(M/B) for DNA segments. The distribution of the average log_2_(M/B) across segments was modeled using a four-component normal mixture model [68]. The EM algorithm [69] was used to estimate the mixing proportion, the mean, and the variance associated with each of the four normal component densities, corresponding to four segment classes (labeled with arrows). Class membership probabilities for each segment were computed using the EM estimates.

In addition to this large region of genome variation on chromosome 6, we expected to identify numerous smaller copy-number variants (CNVs). As seen in the analysis of several well-annotated BACs, there are probes every ∼400 bp in low-copy genomic DNA (Figure 1). CNVs can be discovered by assessing the behavior of adjacent probes to identify segments of DNA that give consistently altered signal from two genomes. The DNAcopy algorithm [34]–[35] was used to identify segments within the CGH dataset with a minimum length of 5 probes (Methods). This resulted in the identification of 53,589 segments that are within a single intra-BAC DNA sequence contig. The distribution of the average log_2_(Mo17/B73) values for each segment was well approximated by a normal mixture model with four components, each corresponding to a different class of segments (Figure 5). Because the component distributions overlap, there is uncertainty about the class membership of each segment. However, it is possible to calculate the probability that any particular segment belongs to a specific class based on the segment's average log_2_(Mo17/B73) value (Methods). Using such probabilities, each segment was classified into its most likely class. This is a relatively permissive approach towards identifying segments. We proceeded to further restrict the results to generate a subset of “stringent” segments that are at least 2,000 bp in length, include at least 10 probes, and, for B73>Mo17 and Mo17>B73 classes, exhibit at least a two-fold difference between average B73 and Mo17 signals (Table 3). The DNA segments from the different classes exhibit different distributions for segment length, probe number/segment and repetitive DNA content (Table 3; Figure S12).

**Table 3:** Characteristics of each category of DNA segment.

All Segments	Type	n	Total # probes	Avg. log2(M/B)	Avg. length (bp)	Avg. probe #	Avg. % repetitive DNA	Avg. # genes	Avg. gene density (per kb)	Avg. % Mo17 coverage
	B73>Mo17_PAV	4,222	52,618	−1.90	7,041	12.5	39.2%	0.6	0.090	7.6%
	B73>Mo17_Int.	3,224	49,222	−1.56	8,859	15.3	57.5%	1.0	0.110	44.3%
	B73>Mo17_CNV	729	10,312	−1.43	8,069	14.1	80.5%	0.6	0.076	96.9%
	B73≈Mo17_SNP	15,976	657,052	−0.43	36,253	41.1	67.6%	2.2	0.059	61.4%
	B73≈Mo17_Int.	9,573	391,681	−0.13	36,076	40.9	67.5%	2.2	0.060	72.3%
	B73≈Mo17	15,536	746,147	0.01	43,122	48.0	72.7%	2.1	0.048	78.2%
	Mo17>B73_CNV	752	11,531	0.82	8,273	15.3	52.8%	1.4	0.167	84.4%
	Unclassified	3,577	110,903	−0.60	26,756	31.0	65.7%	1.6	0.062	53.5%

Stringent segments^a^	Type	n	Total # probes	Avg. log2(M/B)	Avg. length (bp)	Avg. probe #	Avg. % repetitive DNA	Avg. # genes	Avg. gene density (per kb)	Avg. % Mo17 coverage
	B73>Mo17_PAV	1,783	33,773	−1.77	10,688	18.9	44.9%	0.9	0.081	9.3%
	B73>Mo17_Int.	1,542	33,947	−1.46	12,698	22.0	55.2%	1.3	0.104	43.9%
	B73>Mo17_CNV	356	6,846	−1.41	9,878	19.2	84.1%	0.8	0.078	96.7%
	B73≈Mo17_SNP	13,183	637,722	−0.43	41,514	48.4	67.1%	2.5	0.060	62.9%
	B73≈Mo17_Int.	7,526	377,693	−0.13	42,563	50.2	66.6%	2.6	0.061	75.1%
	B73≈Mo17	12,720	726,894	0.01	49,943	57.1	72.5%	2.4	0.048	80.6%
	Mo17>B73_CNV	60	1,110	1.26	6,512	18.5	58.2%	1.1	0.171	94.1%
	Unclassified	2,661	104,579	−0.64	32,619	39.3	65.5%	2.0	0.062	53.1%

a minimum of 10 probes, 2000 bp and 2 fold change between B73 and Mo17 for B73>Mo17 and Mo17>B73 classes.

The B73>Mo17 and Mo17>B73 segments represent DNA sequences that are variable between B73 and Mo17. B73>Mo17 DNA segments could be the result of CNV or differences in genomic content (PAV) between the two lines. In an attempt to distinguish between these two possibilities we determined the proportion of each segment that was non-repetitive and that could be aligned to Mo17 WGS sequence reads. If a large proportion of the segment was found in Mo17 then it is likely that the segment is a CNV, while segments that are missing from the Mo17 WGS likely represent PAVs. The distribution of Mo17 coverage was very different for B73>Mo17 segments compared to the other categories of segments (Figure S13). Over 50% of the B73>Mo17 DNA segments have less than 20% sequence coverage by Mo17 sequences. In the other classes, a majority of segments have >60% coverage by Mo17 WGS. We decided to split the B73>Mo17 segments into three subgroups. B73>Mo17_PAV (present-absent variation) segments exhibit less than 20% coverage by Mo17 WGS reads and are therefore likely present in the B73 genome and absent from the Mo17 genome. B73>Mo17_CNV segments exhibit at least 80% coverage in the Mo17 WGS sequences and are likely examples of CNV. The remaining B73>Mo17 sequences (20%–80% coverage) are denoted as B73>Mo17_Int. (intermediate). As expected, the B73>Mo17_PAV segments have a greater signal difference between B73 and Mo17 than do the B73>Mo17_CNV segments (Table 3).

The segments from the middle two distributions in Figure 5 represent DNA sequences that are present at the same copy number in B73 and Mo17. The segments in the distribution with a peak at log2(Mo17/B73) = −0.43 were classified as B73≈Mo17_SNP while the segments in the distribution with a peak at log2(Mo17/B73) = 0 were simply classified as B73≈Mo17. An additional class, B73≈Mo17_Int. (intermediate), includes DNA sequences that couldn't be definitively classified in either one of these two distributions but had a cumulative estimated probability of membership in these two classes that was greater than 0.8 for these two classes. In general, the B73≈Mo17_SNP, B73≈Mo17_Int. and B73≈Mo17 segments have similar characteristics (Table 3). The B73≈Mo17_SNP and B73≈Mo17_Int. DNA segments have slightly higher levels of signal in B73 than in Mo17 and are likely the result of the inclusion within the segment of several polymorphic probes. This is supported by the slightly higher rates of polymorphic probes or molecular markers within B73≈Mo17_SNP segments than B73≈Mo17 segments (Figure S12B; Table 4).

**Table 4:** # Polymorphic markers within segments.

	# Primers	% polymorphic	% PA^a^
B73>Mo17_PAV	203	75%	83%
B73>Mo17_Int.	288	50%	65%
B73>Mo17_CNV	36	53%	66%
B73≈Mo17_SNP	7,946	28%	35%
B73≈Mo17_Int.	4,484	17%	29%
B73≈Mo17	5,756	6%	27%
Mo17>B73_CNV	9	0%	0%
Unclassified	891	31%	46%
Total	19,613	20%	37%

a The proportion of polymorphic primers that amplify a product in B73 but not in Mo17.

### Characterization of CNVs and PAVs

The segment analysis identified a large number of DNA segments with variation in B73 and Mo17. There are 60 stringent Mo17>B73_CNV segments that are predicted to occur in more copies in Mo17 than in B73. There are 3,681 stringent B73>Mo17 segments including 356 segments that are CNVs and another 1,783 PAV segments that are putative examples of genome content variation.

Several different approaches were used to validate the structural variants identified in this study. The 1,783 stringent B73>Mo17_PAV segments are predicted to be present in the B73 genome but absent from the Mo17 genome. Over 20,000 primer pairs were designed (usually from B73 sequences) and used to perform amplification from B73 and Mo17 genomic DNA. The numbers of primer pairs within each class of segment were determined (Table 4). The proportion of primers that were polymorphic between B73 and Mo17 is much higher for B73>Mo17 segments. The fact that the majority of the B73>Mo17_PAV polymorphic primer pairs only amplify a band in B73 and not in Mo17 confirms that many of these segments are present in the B73 genome and missing in the Mo17 genome. The 356 B73>Mo17_CNV segments are predicted to occur in more copies in the B73 genome than in the Mo17 genome. BLAST searches of 100 stringent B73>Mo17_CNV sequences against the B73 genome find that 92% are present in at least two copies. In comparison, only 7% of the B73≈Mo17 segments have multiple matches within the B73 RefGen_v1. A large proportion (55%) of the B73>Mo17_CNV segments include tandem duplications. This suggests that there are a number of haplotype-specific tandem duplications. The 60 Mo17>B73 segments are predicted to occur in more copies in Mo17 than B73. qPCR was used to assess the copy number for 12 of the 60 Mo17>B73 segments in B73 and Mo17 genomic DNA (Table S2). The increase in copy number in Mo17 relative to B73 was validated for 11 of the 12 segments tested. In three of the cases tested qPCR provides evidence for greater copy number differences than the CGH data, suggesting that the CGH copy number estimates may be conservative. In combination, these approaches provide validation for the three major classes of CNV segments.

**Figure 6:**
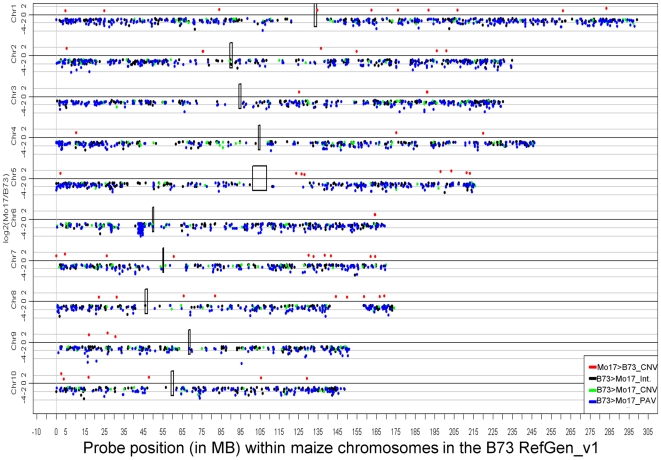
Distribution of CNV and PAV throughout the maize genome. The position and average log_2_(M/B) for each Mo17>B73_CNV, B73>Mo17_CNV, B73>Mo17_I and B73>Mo17_PA segment is plotted for all 10 maize chromosomes. The color-coding indicates the type of segment. The positions of the centromeres [72] are indicated by the black boxes.

The CGH analysis identified hundreds of candidate CNVs and thousands of PAVs. These sequences are spread throughout all ten of the maize chromosomes (Figure 6). The filtered set of 32,540 high quality gene annotations from the MGSP were compared to the stringent DNA segments (Table 5). Using fairly strict criteria (80% of gene sequence is contained within segment sequence) we find approximately 80% of the genes are located within the stringent segments. Almost 600 of these genes are located in the B73>Mo17 or Mo17>B73 segments, including 180 gene models located within B73>Mo17_PAV segments and another 50 gene models located within CNV segments. These genes within the PAV and CNV type segments include many different annotations and are not enriched for putative uncharacterized proteins. Interestingly, the proportion of genes with a paralog (defined as >85% identity and coverage) is higher for the B73>Mo17 segments (Table 5). A portion of the genes within these segments are queried by the existing 17K maize Affymetrix microarray. The proportion of genes that are differentially expressed (in B73 and Mo17 seedling tissue; data from [11]) is much higher for B73>Mo17 and Mo17>B73 segments than for B73≈Mo17 classes (Table 5). As expected, the B73>Mo17 segments are enriched for genes with higher expression in B73 than in Mo17 and the Mo17>B73 segments are enriched for genes with higher expression in Mo17.

**Table 5:** Genes in stringent segments.

Type	n	Avg. length (bp)	# FGS genes^a^	Genes per segment	% of genes with paralog	# Affymetrix genes^b^	%DE genes^c^	%DE with B>M^d^
B73>Mo17_PAV	1,783	10,688	180	0.10	63.4%	36	69%	92%
B73>Mo17_Int.	1,542	12,698	360	0.23	61.6%	68	56%	92%
B73>Mo17_CNV	356	9,878	41	0.12	62.5%	7	71%	100%
B73≈Mo17_SNP	13,183	41,514	10491	0.80	50.0%	3,347	24%	49%
B73≈Mo17_Int.	7,526	42,563	5748	0.76	49.3%	1,806	18%	48%
B73≈Mo17	12,720	49,943	7831	0.62	49.7%	2,306	12%	33%
Mo17>B73_CNV	60	6,512	9	0.15	54.5%	7	100%	0%
Unclassified	2,661	32,619	1364	0.51	55.4%	361	31%	66%

a The FGS refers to the filtered gene set of high-quality annotations produced by the MGSP.

b Number of genes on Affy platform that are expressed in B73 or Mo17 seedling tissue.

c Percent of genes that are differentially expressed (q<0.05).

d Percent of differentially expressed genes that are expressed at higher levels in B73 than in Mo17.

## Discussion

There is wide-spread appreciation for the high level of diversity within the maize species [8],[12],[31]. This diversity is critical for breeders to select for novel agronomic traits and is important for heterosis. The availability of a reference genome sequence for one inbred (B73; Schnable et al., in press) coupled to CGH technology, has provided the opportunity to study the structural variation present between two inbred lines, B73 and Mo17. The extensive structural variation between B73 and Mo17 includes copy number variation (CNV) and present-absent variation (PAV). However, despite the high level of variation genome-wide, there are many regions of the genome that have little or no variation. We will discuss the types of variation observed throughout the maize genome as well as the implications of this variation for phenotypic diversity and heterosis.

### Low diversity regions in a highly polymorphic species

It is tempting to assume that all genomic regions are different in these two lines. However, by assessing the levels of variation along the B73 RefGen_v1 it quickly becomes obvious that this variation is not randomly distributed. We identified a number of large regions (>1 Mb) that have little or no variation. The fact that these regions co-localized with chromosomal regions that lack genetic markers that exhibit polymorphisms in the Intermated B73xMo17 (IBM) mapping population [14],[36] demonstrates that these marker-depleted regions are simply due to low/no diversity between the two parents of the mapping population. In general, almost all of the large low diversity regions occur within regions of low recombination frequency. This could contribute to the inheritance of large chromosomal regions that are identical-by-descent. The centromeres of most chromosomes are located within or at one end of low-diversity regions.

Several groups have assessed molecular diversity in maize populations in studies designed to identify the targets of domestication and/or selection in maize [37]–[39]. We noticed that two of the genes known to have been targets of selection or domestication, *y1* [40] and *tb1* [41] are located within large low diversity regions. In addition, a 1 Mb region on chromosome 10 with evidence for a selective sweep [42] also occurs in a region with low levels of structural variation. The chromosomal positions of 42 genes with evidence for selective sweeps [37]–[38] were compared with the level of structural variation. Nearly half of these genes (20/42) were located within large blocks of low diversity identified in this study. This finding is consistent with the hypothesis that many putative selection genes identified by virtue of their limited sequence diversity were not actual targets of selection but simply happen to be located within large blocks of reduced variation, some of which may have arisen via selective sweeps.

### Frequency of structural variation in maize genome

We have identified thousands of examples of structural variation between the B73 and Mo17 genomes. The term structural variant is used to describe both sequences that are present in both individuals but have different copy numbers (copy number variants; CNV) and sequences that are present in one individual but absent in another (presence-absence variant; PAV). The unknown order and orientation of intra-BAC DNA sequence contigs will potentially lead to an over-estimation of the number of structural variation events by splitting some events into two different segments. However, it will also lead to an under-estimation of the number of events due to the fact that some smaller structural variant events which will not have enough sequence or probes on either side of a contig border to be called. We found that the 3,789 stringent B73>Mo17 or Mo17>B73 structural variants represent a minimum of 2,056 unique events (must be separated from nearest structural variant by >200,000 bp).

Differing probe densities, algorithms and statistical criteria complicate comparisons of rates of structural variation among organisms [5]. However, it is quite clear that the maize genome has a high rate of structural variation compared with other species. In the human, rat, dog, mouse, macaque and chimpanzee genomes the average number of CNVs between two individuals is between 15 and 75 [43]–[48]. A high resolution study of eight human genomes [49] revealed only several hundred insertions and deletions, including CNV and PAV sequences, in the comparison of any two human genomes. In contrast, even after very stringent filtering we identified >3,700 CNV or PAV sequences that represent at least 2,000 events between these two maize genomes. This likely represents a very conservative estimate of the true number of CNV and PAV events in the maize genome. Previous analyses of BAC libraries have also found significant differences in genome content [25]. This high level of structural variation with frequent changes in genome content is reminiscent of the high level of variation observed in the *E. coli* genome [50]–[51], but is without precedent among higher eukaryotes. As the levels of structural variation are assessed for other species it will be interesting to determine whether maize has an unusually high level of variation relative to other plants and animals.

### Mechanisms and impact of CNV

This study identified >400 putative CNVs between B73 and Mo17. A combination of genome homology searches and qPCR suggests that many of these sequences represent actual CNVs. There is evidence that these CNV can be the result of tandem duplications or duplications dispersed throughout the genome. There are a large number of tandem duplications in the maize genome [32]; some of these are NIPs [29] and 5% of these exhibit CNV in our data (Y. Kai, P. Schnable, unpublished data). This suggests that some of the differences in copy number between B73 and Mo17 are due to haplotype-specific tandem duplication events. Alternatively, some of the CNVs may be caused by duplication to non-allelic positions. Differences in genome content at the *bz1* locus [23] actually represent a CNV event in which both genotypes have copies of a sequence at a shared position and one of the genotypes has one or more additional copies at a non-shared location [26]. Many of these CNV are likely the result of Helitron-mediated movement of gene fragments [12], [25]–[26]. There is also evidence that tandemly duplicated gene families such as zeins [52] or disease resistance genes [53] exhibit differences in copy number for different haplotypes, possibly as the result of recombination-based mechanisms as have been analyzed in detailed by Yandeau-Nelson et al. [54].

Previous studies have suggested a high rate of near identical paralogs (NIPs) in the maize genome [29]. It is likely that the formation (or removal) of NIPs may have been haplotype specific. There are 50 genes from the MGSP's filtered gene set within our stringently called CNVs. By relaxing our criteria only slightly, we identify CNV segments that contain 558 genes (Table S3). As most gene fragments were successfully removed from the MGSP's filtered gene set [32], these genes within CNV are likely to include functional genes. Because 12 of the 14 CNV genes that were assayed exhibit variable gene expression levels in B73 and Mo17 seedlings, these genic CNV may contribute to phenotypic diversity.

Many maize alleles that are known to be epigenetically regulated exhibit allelic variation for tandem repeats. There is allelic variation in the tandem duplication of coding regions at the *p1*, *c2*, and *r1* loci that exhibit epigenetic regulation [54]–[56]. In addition, there is evidence for allelic variation in the copy number of a non-genic sequence ∼100 kb upstream of the *b1* gene that controls expression and paramutation [57]. It is possible that the high rates of CNV in both genes and other low-copy sequences contribute to high rates of expression variation and epigenetic regulation in maize.

### Widespread genome content differences

In addition to the hundreds of CNV detected between B73 and Mo17 we also noted thousands of sequences that account for over 20 Mb of DNA that are present in the B73 genome and absent in the Mo17 genome. It is quite unexpected to find such a large number of sequences that are present within one haplotype of a species and missing from another. These include extreme examples such as the 2 Mb region on chromosome 6 as well as many smaller B73>Mo17_PAV sequences. Following the initial discovery of PAV sequences we sought to determine whether these PAVs included genes and to estimate the number of genes affected by PAV. Many PAV segments include genes contained within the MGSP filtered gene set. It is important to note that the MGSP filtered gene set was rigorously filtered to remove gene fragments and sequences with homology to transposable elements [32]. While it is possible that a subset of the PAV sequences may represent novel, uncharacterized transposable elements, it is clear that numerous genes are present in the PAV sequences. Several examples of putative genes that that are unlikely to represent transposable elements but that exhibit PAVs include GRMZM2G390498 (putative superoxide dismutase), GRMZM2G066290 (putative pyruvate kinase), GRMZM2G139160 (C2H2 zinc finger protein) and GRMZM2G382393 (putative auxin efflux carrier).

It is, however, difficult to determine the exact number of genes affected by PAV because this number is strongly influenced by the stringency used to identify PAV sequences and by the criteria used to identify genes within the PAV sequences. Using quite strict criteria for identifying segments and genes within the segments, the PAVs include 180 genes from the MGSP's filtered gene set (Table S4) and the B73>Mo17_Int. segments include another 360 genes. These 180 and 360 genes all have at least 80% of the gene length included in the PAV sequence implying that these are full-length genes and not simply examples of PAV for gene fragments of the type reported by Morgante et al. (2005). A more permissive approach (Table S3) finds as many as 473 genes within the B73>Mo17_PAV and another 797 genes within the B73>Mo17_Int. seqments. The very conservative estimate of gene number, 180, or the more permissive estimate of gene number within PAV sequences, 1,270 (473+797), account for 0.5% or 4.0% of the genes within the MGSP's set of filtered genes, suggesting that PAV affects a significant portion of maize genes.

These present-absent sequences are spread throughout the B73 genome. These events differ in a significant way from those observed by Fu and Dooner [23] who detected copy number differences within small gene families. In contrast PAVs are low- or single-copy DNA sequences that occur in B73 and are not present anywhere in the Mo17 genome. Many these genes are expressed and as expected, the majority is expressed in B73 but not in Mo17. In addition, over 1/3 of the gene models within PAV sequences do not contain similar sequences located elsewhere in the B73 genome (Table S3). This suggests that the some examples of PAV (those with paralogs) may be functionally complemented by another gene but that a significant portion of the PAV sequences do not have a functional complement elsewhere in the maize genome.

The high level of PAV sequences between B73 and Mo17 may reflect ancient haplotype variation or more recent genomic rearrangements. We assessed the prevalence of 85 B73>Mo17_PAV segments in 22 other inbred lines (listed in Figure 4) using IDP primers [14]. Interestingly, all 85 of these segments are detected in at least two of the other inbred lines. The majority of these segments (53/85) are present in 30–70% of the other lines. The common presence/absence of these segments suggests that they often reflect relatively old events and not novel, inbred-specific, events.

While there is substantial phenotypic diversity between B73 and Mo17 even non-biologists quickly recognize both as corn plants. It is surprising that these inbreds can tolerate such a high level of genome content variation and still develop as “normal” corn plants. It is likely that deleterious PAVs have been strongly selected against. Maize is normally an out-crossing species. Due to inbreeding depression, many of the first generation inbreds produced by breeders in the early part of the last century were drastically reduced in fitness, incapable of reproducing or even inviable. Those first-generation inbreeds that could be propagated were intercrossed to produce the second and subsequent generations of inbreds which comprise the commercial gene pool of maize. Hence, PAVs with strong effects on fitness would likely have been purged from the commercial gene pool. It will therefore be of great interest to explore the PAV content of landraces of maize that have not been subjected to the inbreeding bottleneck.

### Potential impact of structural variation on phenotypic diversity and heterosis

The frequent CNV and PAV observed among maize inbreds may contribute to the high levels of phenotypic diversity and plasticity observed in maize. CNV and PAV can have significant contributions to phenotype. There is evidence that tandem duplications may be important for the evolution of traits such as disease resistance [58]. In addition, the variation in copy number may allow for the evolution of novel expression patterns. There is evidence that strong artificial selection on specific anthocyanin coloration patterns has often led to the formation of complex alleles with tandem duplications [56], [59]–[60]. The presence of many CNV and PAV events provides opportunity for selection. As different structural variants are combined through breeding there is opportunity for novel trans-interactions and for formation of novel alleles through unequal crossing over. Long-term selection experiments (>100 years) have continued to make progress on quantitative traits [30] and it is possible that the genomic variation of maize provides source material to generate novel alleles.

The high levels of structural variation detected in this study and high levels of heterosis observed in certain hybrids of maize may be linked. Heterosis (the superior performance of a hybrid relative to its inbred parents) has pronounced and widespread effects on many traits. The high frequency of genome content differences suggests a large number of linked content differences. In this study we identify several thousand sequences that are present in B73 but missing in Mo17. If we assume that there are an equivalent number of sequences that are present in Mo17 but absent in B73 we would expect nearly 4,000 genome content differences distributed throughout the B73 and Mo17 genomes. The finding of single-copy, expressed PAVs among maize inbreds demonstrate that it will be important to obtain the genome sequences of a number of inbred lines to identify the full complement of genes present within the maize species. The large number of potential combinations of PAV sequences in hybrids also provides the opportunity for novel gene complements in hybrids relative to the parental lines. Previous analyses of gene expression in B73, Mo17 and the F_1_ hybrid identified a number of genes that are expressed in one parent but not the other [11]. Interestingly, all of these genes are expressed in the hybrid leading to a larger number of transcripts in hybrids than in the inbred parents. In addition, the combination of inbred-specific sequences in the hybrids provides opportunities for novel trans-interactions that would not occur in either parent potentially leading to non-additive expression levels [10],[61]. Hence, further explorations of genomic variation among maize lines may lead to opportunities to elucidate the mechanisms of heterosis.

## Methods

### Microarray design

An oligonucleotide microarray was designed by Roche NimbleGen to perform comparative genomic hybridization (CGH) of maize inbreds (Copies of this design may be acquired by ordering: 080418_zea_mays_B73_CGH_HX1). A set of 14,423 maize BACs (downloaded March 2008) was used to design isothermal probes, varying in length from 45 bp to 85 bp and with a target Tm of 76C at a fixed interval of 50 bp. Probe sequences were repeat-masked by calculating the average 14-mer frequency for each probe, based on a frequency table generated from the complete set of BAC sequences available as of that date, and removing probes with an average 14-mer frequency higher than 400. Probe uniqueness was determined by comparing each probe to B73 RefGen_v1, using SSAHA (http://www.sanger.ac.uk/Software/analysis/SSAHA/) with a step-size of 1, nmer-size of 12 and a minimum match length of 33 bp. Up to five insertions/deletions were allowed in each match. Probes with < = 15 close matches in the genome were included in the array design. Median final probe spacing was 450 bp. It should be noted that this set of probes was designed to facilitate sequence capture [62]; if NimbleGen's CGH probe design criteria had been utilized it is likely that the choice of probes would have been slightly different.

### Probe annotations

The sequences of CGH probes were aligned to the B73 RefGen_v1 (Schnable et al., in press); >90% of the probes (1,977,283/2,124,029) could be mapped with 100% identity and coverage (Figure S1). These include probes with a single match to the B73 RefGen_v1 as well as probes with multiple perfect matches (Figure S1). The remaining 146,746 probes (either imperfect matches or not found in the B73 RefGen_v1) had very low hybridization signals suggesting that they were likely artifacts created by using unfinished BAC for probe design and were therefore omitted from all subsequent analyses. The probes that could be mapped to the B73 RefGen_v1 were further annotated for repetitiveness, for gene annotation and for sequence conservation with Mo17. The “repetitiveness” of each of the probes was classified using a series of repeat filters. The ∼3% (54,791) that match at least five locations in the B73 genome with >97% identity and coverage were designated as “multi-copy”. The ∼25% (530,314) that aligned to five or more genomic locations at reduced stringency (>90% identity and coverage) were designated as “crosshyb” probes. There were also 63,792 probes that match the ISU cereal repeat database (http://magi.plantgenomics.iastate.edu/) but did not meet the criteria for designation as multi-copy or icicle probes. Generally, the different classes of repetitive probes exhibit similar behavior and we will therefore refer to multi-copy, icicle and cereal repeat probes as “repetitive probes”. The remaining 1,461,771 probes were designated as non-repetitive. The location of each probe relative to genic sequences was determined through comparisons to gene models provided by the MGSP and were assigned to the following classes: exon, exon-intron (crosses exon/intron border), intron, 5′ (within 2,000 bp 5′ of start site), 3′ (within 2,000 bp 3′ of the gene), intergenic (more than 2 kb from nearest gene). The conservation of sequences of individual probes to the Mo17 genome was classified via alignments to the 42,206,664 Mo17 WGS sequences provided by Daniel Rohskar from the DOE's Joint Genome Institute. Each probe was classified as perfect match (100% identity and coverage), highly conserved (>97% identity and coverage, not perfect match), conserved (97–90% identity and coverage, poorly conserved (75–90% identity and 70%–90% coverage not highly conserved) or no match (all other probe).

### Microarray hybridizations

Total genomic DNA isolated from two-week-old etiolated seedlings of maize inbreds B73 and Mo17 were labeled and hybridized following the methods described in Selzer et al. [63] and Roche NimbleGen's CGH user's guide (see manufacture's User guide). In short, 1 ug of DNA was labeled using either 5′ Cy3 or Cy5-labeled Random Nonamers (TriLink Biotechnologies). DNA was incubated for 2 hours at 37°C with 100 units (exo-) Klenow fragment (NEB) and dNTP mix (6 mM each in TE; Invitrogen). The labeled samples were then precipitated with NaCl and Isopropanol and then rehydrated in 25 µl of VWR H20. 34 µg of test and reference samples were combined in a 1.5 ml tube and dried down by SpeedVac. Samples were resuspended in 12.3 µl of H20 and 31.7 µl of Roche NimbleGen Hybridization Buffer (Roche NimbleGen Inc.) and incubated at 95°C. The combined and resuspended samples were then hybridized to the array for 60–72 hours at 42°C degrees with mixing. Arrays were washed using Roche NimbleGen Wash Buffer System and dried using the NimbleGen Microarray Dryer (Roche NimbleGen, Inc). Arrays were scanned at 5 µm resolution using the GenePix4000B scanner (Axon Instruments). Data was extracted from scanned images using NimbleScan 2.4 extraction software (Roche NimbleGen, Inc.), which allows for automated grid alignment, extraction and generation of data files. In our experimental design we had seven replicates of B73 (one with Cy3 and six with Cy5) and seven replicates of Mo17 (six with Cy3 and one with Cy5). Images were processed and spatial normalization of data within the array was conducted according to NimbleGen's standard protocol. Due to the fact that our array was designed using B73 genomic sequence and the high rate of polymorphism between B73 and Mo17, the CGH data violated the assumption for the regularly used Q-spline normalization to make two channels of hybridization intensities comparable (see supplemental figures for further information on normalization issues and solutions). We used a subset of 840,289 probes that were known to have identical sequence in B73 and Mo17 (based on B73 BAC sequences and Mo17 WGS data) as a control set to obtain a best-fitting cubic spline function [64], assuming that most of these probes should have the same hybridization intensities after normalization. The spline function was then globally applied for all probes to normalize the two channels. After within-chip normalization, linear model analyses using LIMMA [65]–[66] were conducted for the data from all microarrays. The linear model for each probe included effects for dyes and genotypes, and p-values were calculated to test for a signal difference between Mo17 and B73 genotypes as part of each linear model analysis. The p-values were converted to q-values which were used to control the false discovery rate as described by Storey and Tibshirani [67].

### Segmentation analysis

A segmentation analysis was performed using DNAcopy [34]–[35]; with tune default parameter alpha = 0.05, trim = 0.05) to identify groups of probes that exhibit similar deviation from a log2(M/B) ratio of zero. These probes identify segments of DNA that have DNA sequence polymorphisms, altered copy number, or presence/absence in the two genotypes. The ordering and orientation of intra-BAC DNA sequence contigs, and therefore probe sequences, within a BAC has not been fully determined for most of the BACs. Consequently, the resulting segment predictions were split at intra-BAC DNA sequence contig boundaries following the DNAcopy analysis. All segments were assigned a unique identification and the average log2(M/B) for all probes within the segment was determined. The distribution of the average log2(M/B) across segments was modeled using a four-component normal mixture model [68]. The EM algorithm [69] was used to estimate the mixing proportion, the mean, and the variance associated with each of the four normal component densities, corresponding to four segment classes. Class membership probabilities for each segment were computed using the EM estimates. Each segment was then classified into one of the four classes if its most likely class was greater than 0.8. The segments were further filtered to remove all segments that contain fewer than 10 probes or 2000 bp of sequence to produce a set of stringent segments. The underlying sequence of these segments was obtained by parsing the segment data to produce a sequence that spanned the full segment. These segments were then further annotated by comparisons to repeats, gene predictions and Mo17 sequence. In order to classify a gene within a segment we required that 80% of the gene sequence be within the segment sequence.

### Analyses of gene expression

Gene expression information was obtained from several different sources. The Affymetrix data was obtained from 11-day old seedlings (GEO: GSE8174; [11]) and cDNA microarray expression data was obtained 14-day old seedling tissue (GEO_ GSE3733; [10]). RNA-Seq data was obtained from B73 shoot apical meristem tissue (SAM) isolated as described by Ohtsu et al. [70]. A pool of RNA sample from L1 of 13 SAMs and a pool of RNA samples from L2 of 13 SAMs were extracted followed by RNA amplification and synthesis of double-stranded cDNA according to previous procedures [70]. The libraries were sequenced on the Solexa 1G Genome Analyzer at Canada's Michael Smith Genome Sciences Centre. Each library was sequenced using 2 lanes on a Solexa flow cell. The resulting Solexa reads were aligned to maize gene models (http://www.maizesequence.org) with the short read aligner NOVOALIGN (http://www.novocraft.com) using 32 bases. The low quality bases located at the end of reads were trimmed off by the program and only reads that mapped uniquely to the genome with a maximum of two mismatches including insertion/deletion (indel) across 32 bases were used for subsequent analyses. The reads uniquely mapped to genome were projected to gene models (release 4a.53).

### qPCR validation of Mo17>B73 CNV

Primers were designed for 12 Mo17>B73_CNV segments (Table S2). 20ng of three biological replicates of genomic DNA isolated from B73 or Mo17 seedlings was amplified with Applied Biosystems SYBR Green 2× PCR Master Mix (Applied Biosystems) using an Applied Biosystems 7900HT Real-Time PCR System in a 20µl reaction volume. Two technical replicates were performed for each sample. The average cycle threshold (Ct) values were determined for the technical replicates. The relative copy number was determined by comparing the Ct value for the test primer set to three different genomic controls known to be present in one copy in each genome.

## Supporting Information

Figure S1 Flow-chart detailing the mapping of probe sequences to the B73 RefGen_v1. Probes with 100% identity and coverage were retained for analyses.
(0.11 MB PPT)

Figure S2 Significant hybridization differences are due to structural variation. The B73 and Mo17 sequences for two portions (A and B) of the bz1 locus (sequenced and annotated by Fu and Dooner 2002 and Brunner et al., 2005) were aligned using Vista (Frazer et al., 2004) which displays the percent identity as a sliding window of 100 bp (y-axis is 50% to 100% identity). The location of genes (indicated by light blue sequences in the alignment) and repeat elements (the color-coded track right above the alignments; retrotransposons are shaded pink and transposons are shaded orange) are shown above the VISTA alignment. The log2(Mo17 signal/B73 signal) is shown for each probe in this region. The red probes exhibit significantly different (q<0.0001) signal in B73 and Mo17. The repetitive annotation is shown as a track below the log signal (blue are repetitive probes and black are non-repetitive probes). The blue line indicates a segment with altered hybridization that was identified using DNAcopy. Note that these annotations are based on the genome-wide analysis, not detailed analyses of these regions. The last four probes in (A) and the fist four probes in (B) occur in regions where Mo17 does not have similar sequence at the allelic position but do not show significant differences in hybridization. This suggests that there are examples of sequences that are present in Mo17 but at a non-allelic position.
(0.45 MB PPT)

Figure S3 Density plots of sample chip signal intensity before and after global q-spline normalization. The distribution of B73 (red) and Mo17 (green) signals in raw data (A). Note that the distribution of signals is quite different for the two genotypes. In (B) the raw data were normalized using the global q-spline approach. This approach altered the distribution of signals such that the two genotypes exhibit similar distributions. In (C), the data were normalized using the B = M probes as a training set prior to global q-spline normalization. This approach preserves the original distributions of the signals for the two genotypes.
(0.07 MB PPT)

Figure S4 Alterations of the distribution of log2(M/B) values following different normalization approaches. In (A) a global q-spline normalization was applied to the data. The resulting log2(M/B) values exhibit a non-uniform distribution that is centered at 0.3 and a long tail towards negative log2(M/B) values. However, when the “B = M” probes are used as a training set prior to normalization, the distribution of values is centered near zero. This suggests the using the “B = M’ probes can provide a mechanism for appropriate normalization of this dataset.
(0.06 MB PPT)

Figure S5 Distribution of hybridization values in B73 and Mo17. (A) A volcano plot was used to show the distribution of q values (y-axis) relative to the log2(Mo17/B73) ratios (x-axis). Note that there are more significant probes with a negative log2 (M/B) value (upper left) than probes with a positive log2 (M>B) value. (B) The MA plot shows that there is a substantial bias towards low signal probes with a -M value.
(0.07 MB PPT)

Figure S6 Volcano and MA plots for classes of repetitive probes. (A) The multi-copy repeat probes (at least 5 copies of >97% identity and coverage) are shown in blue. Many of these probes have high hybridization signals. (B) The crosshyb repeat probes (at least five genomic loci with 90% identity and coverage) are shown in red. These probes rarely show significant differences and have a range of different hybridization values. (C) The cereal repeat probes (similar to sequences in ISU cereal repeat database) are shown in yellow.
(0.38 MB PPT)

Figure S7 Repetitive probes rarely report variation in B73 and Mo17. The chromosomal distribution (x-axis) is shown for each class of repetitive probe relative to the log2(Mo17/B73) (y-axis). (A) The multi-copy repeat probes (at least 5 copies of >97% identity and coverage) are shown in blue and all other probes are shown in gray. (B) The crosshyb repeat probes (at least copies that have 90% identity and coverage) are shown in red and all other probes are shown in gray. (C) The cereal repeat probes (similar to sequences in ISU cereal repeat database) are shown in yellow and all other probes are shown in gray.
(0.54 MB PPT)

Figure S8 Annotation of probes that exhibit significant (q<0.0001) variation in hybridization to B73 and Mo17 genomic DNA. (A) The percentage of all probes, B73>Mo17 probes and Mo17>B73 probes that are classified as non-repeat, multi-copy, icicle or cereal repeats. (B) For the same sets of probes, the conservation of probe sequence in Mo17 was assessed. (C) The location of significant probes relative to the MGSC working set of genes was also assessed. Each probe was classified as exon, other genic (including intron, UTR, or junctions), or non-genic.
(0.10 MB PPT)

Figure S9 Volcano and MA plots for differing levels of conservation in Mo17 sequence. Each probe was compared to the Mo17 454 WGS sequence (provided by the Joint Genome Institute) and classified as perfect match (100% identity and coverage), highly conserved (>97% identity and coverage), conserved (>90% identity and coverage), poorly conserved (>75% identity and 70% coverage) or no match. The distribution of signals and variation for each type of probe are shown using volcano plots and MA plots. The pie chart shows the relative proportion of each type of probe.
(0.50 MB PPT)

Figure S10 Rates of variation and chromosomal distribution of probes with different levels of B73-Mo17 sequence conservation. The boxes indicate the positions of the centromeres (from Wolfgruber et al.[72]).
(0.18 MB PPT)

Figure S11 Genomic regions of low (A) or high (B) levels of structural variation. The log2(Mo17/B73) hybridization intensities are plotted for a region on chromosome 8 (A) with low levels of probes that detect structural variation. In (B) the hybridization intensities are plotted for all of the probes within a region on chromosome 6 with high levels of probes that detect structural variation.
(0.15 MB PPT)

Figure S12 Annotation of probes that are within stringent segments that are present only in B73, or are higher in copy number in B73 or in Mo17. (A) The proportion of probes within stringent segments that are classified as non-repeat, multi-copy, icicle or cereal repeats. (B) For the same sets of probes, the conservation of probe sequence in Mo17 was assessed. (C) The location of probes relative to genes was also assessed. Each probe was classified as exon, exon-intron, intron, 5′ 2000bp or 3′ 2000bp.
(0.19 MB PPT)

Figure S13 Distribution of Mo17 coverage for DNA segments in each category. The proportion of stringent segments with the specified coverage by the Mo17 454 WGS reads are specified for each category. Note that the coverage statistics are the proportion of non-repetitive bases within the DNA sequence that are covered by Mo17 WGS sequence.
(4.44 MB PPT)

Table S1 IDPs within B73-specific chromosome 6 region.
(0.04 MB XLS)

Table S2 qPCR validation of Class 4 CNVs.
(0.03 MB XLS)

Table S3 Number of genes included within CNV and PAV segments.
(0.03 MB XLS)

Table S4 Annotation of 180 filtered genes located within PAV segments.
(0.08 MB XLS)
