# Supplementary figures and images for: Maize Inbreds Exhibit High Levels of Copy Number Variation (CNV) and Presence/Absence Variation (PAV) in Genome Content
Source: PLoS Genet. 2009 Nov 20;5(11):e1000734. doi: 10.1371/journal.pgen.1000734 (PMC2780416; doi:10.1371/journal.pgen.1000734)

## Slide 1
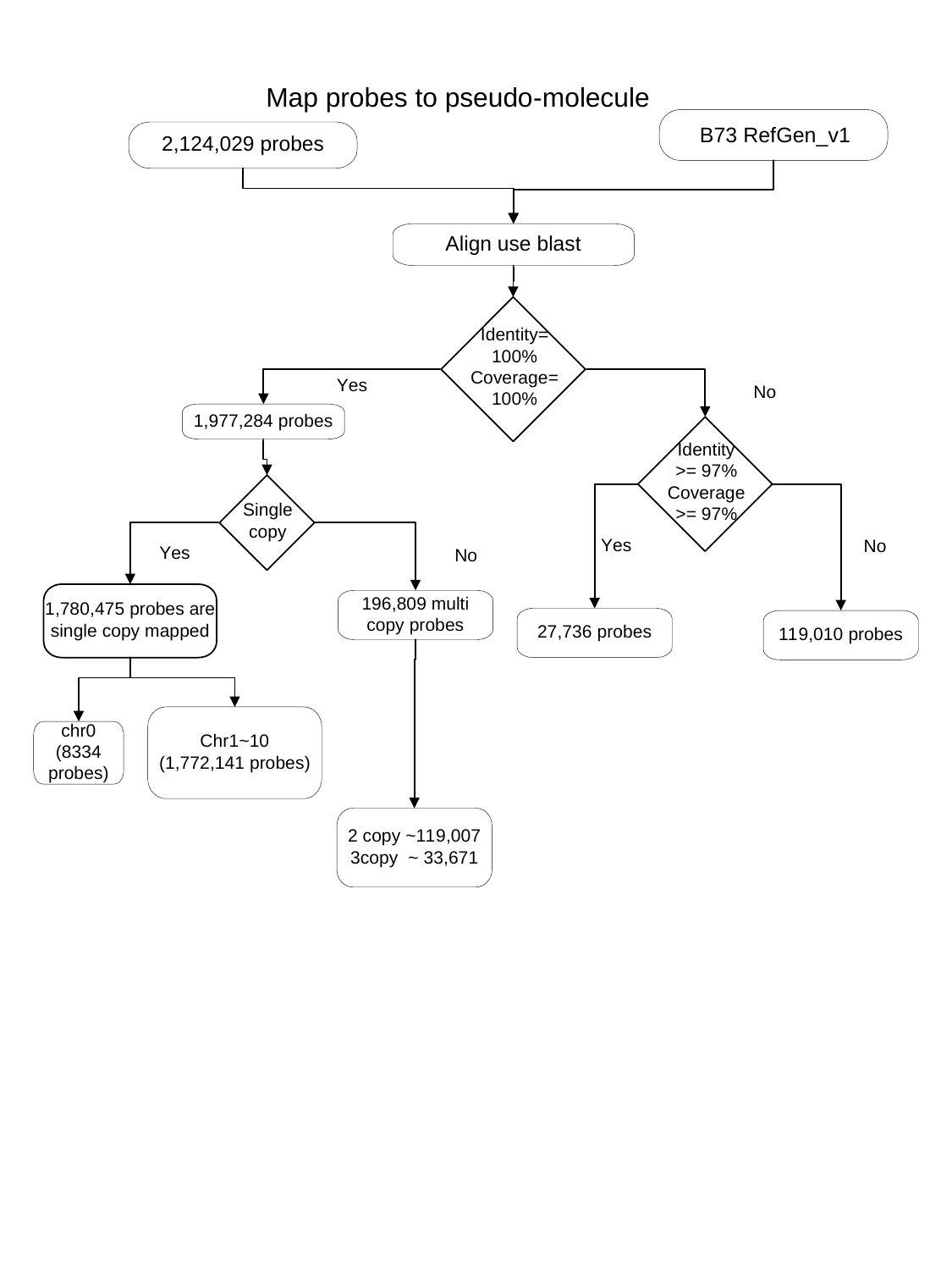

B73 RefGen_v1

Supplement: Figure S1 — Flow-chart detailing the mapping of probe sequences to the B73 RefGen_v1. Probes with 100% identity and coverage were retained for analyses. (0.11 MB PPT) [file pgen.1000734.s001.ppt]

## Slide 1
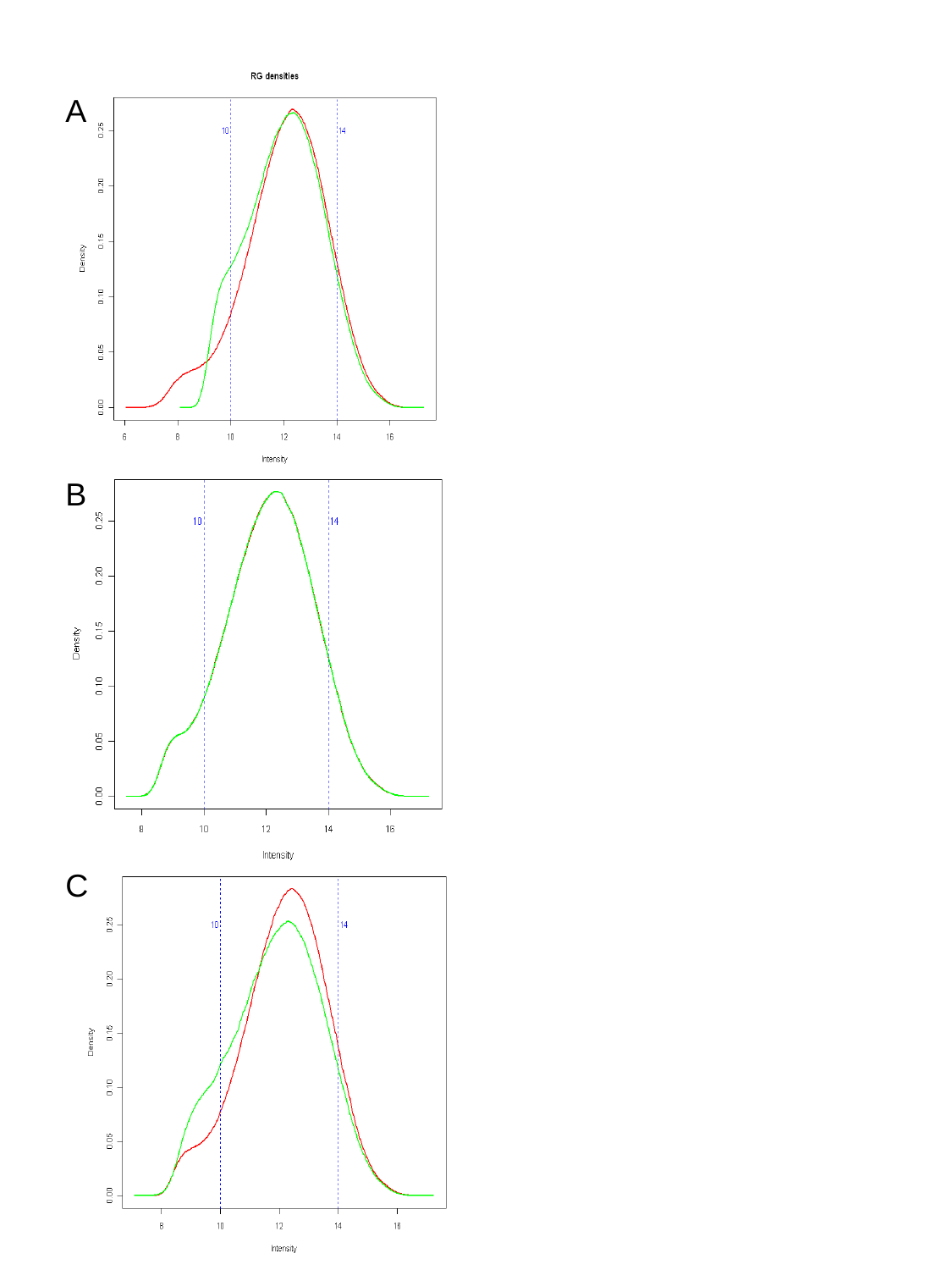

A
B
C

Supplement: Figure S3 — Density plots of sample chip signal intensity before and after global q-spline normalization. The distribution of B73 (red) and Mo17 (green) signals in raw data (A). Note that the distribution of signals is quite different for the two genotypes. In (B) the raw data were normalized using the global q-spline approach. This approach altered the distribution of signals such that the two genotypes exhibit similar distributions. In (C), the data were normalized using the B = M probes as a training set prior to global q-spline normalization. This approach preserves the original distributions of the signals for the two genotypes. (0.07 MB PPT) [file pgen.1000734.s003.ppt]

## Slide 1
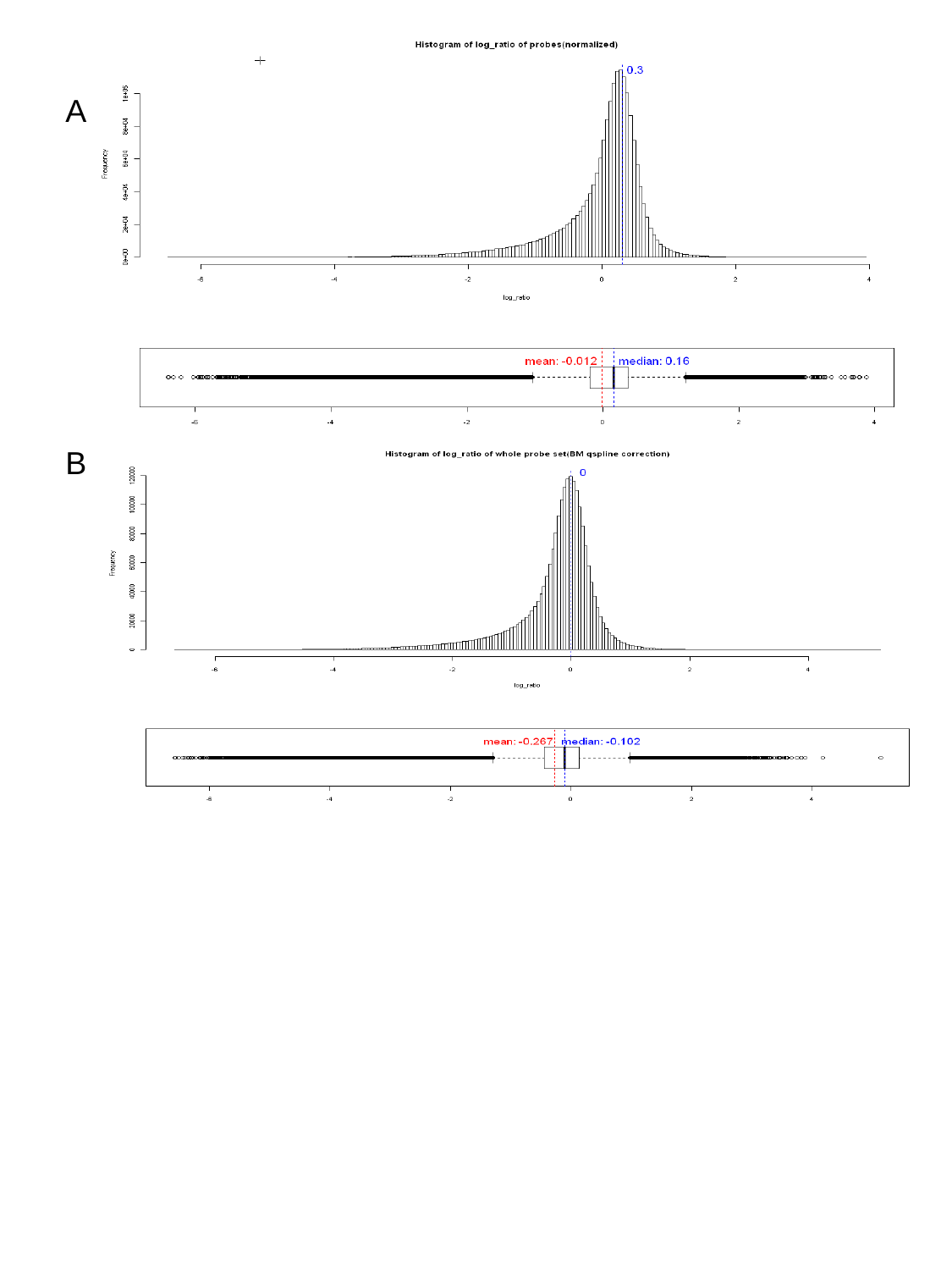

A
B

Supplement: Figure S4 — Alterations of the distribution of log2(M/B) values following different normalization approaches. In (A) a global q-spline normalization was applied to the data. The resulting log2(M/B) values exhibit a non-uniform distribution that is centered at 0.3 and a long tail towards negative log2(M/B) values. However, when the “B = M” probes are used as a training set prior to normalization, the distribution of values is centered near zero. This suggests the using the “B = M’ probes can provide a mechanism for appropriate normalization of this dataset. (0.06 MB PPT) [file pgen.1000734.s004.ppt]

## Slide 1
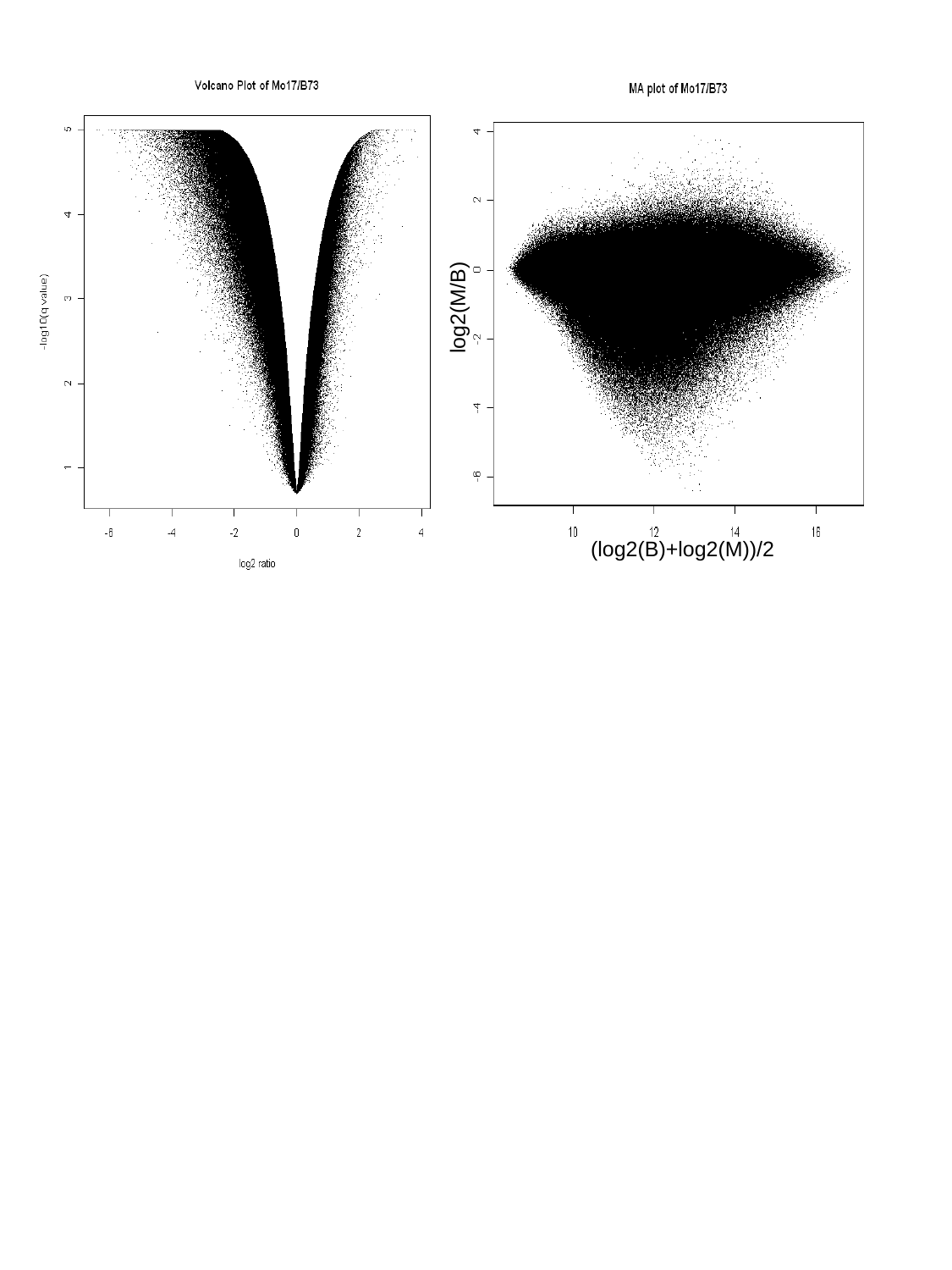

log2(M/B)
(log2(B)+log2(M))/2

Supplement: Figure S5 — Distribution of hybridization values in B73 and Mo17. (A) A volcano plot was used to show the distribution of q values (y-axis) relative to the log2(Mo17/B73) ratios (x-axis). Note that there are more significant probes with a negative log2 (M/B) value (upper left) than probes with a positive log2 (M>B) value. (B) The MA plot shows that there is a substantial bias towards low signal probes with a -M value. (0.07 MB PPT) [file pgen.1000734.s005.ppt]

## Slide 1
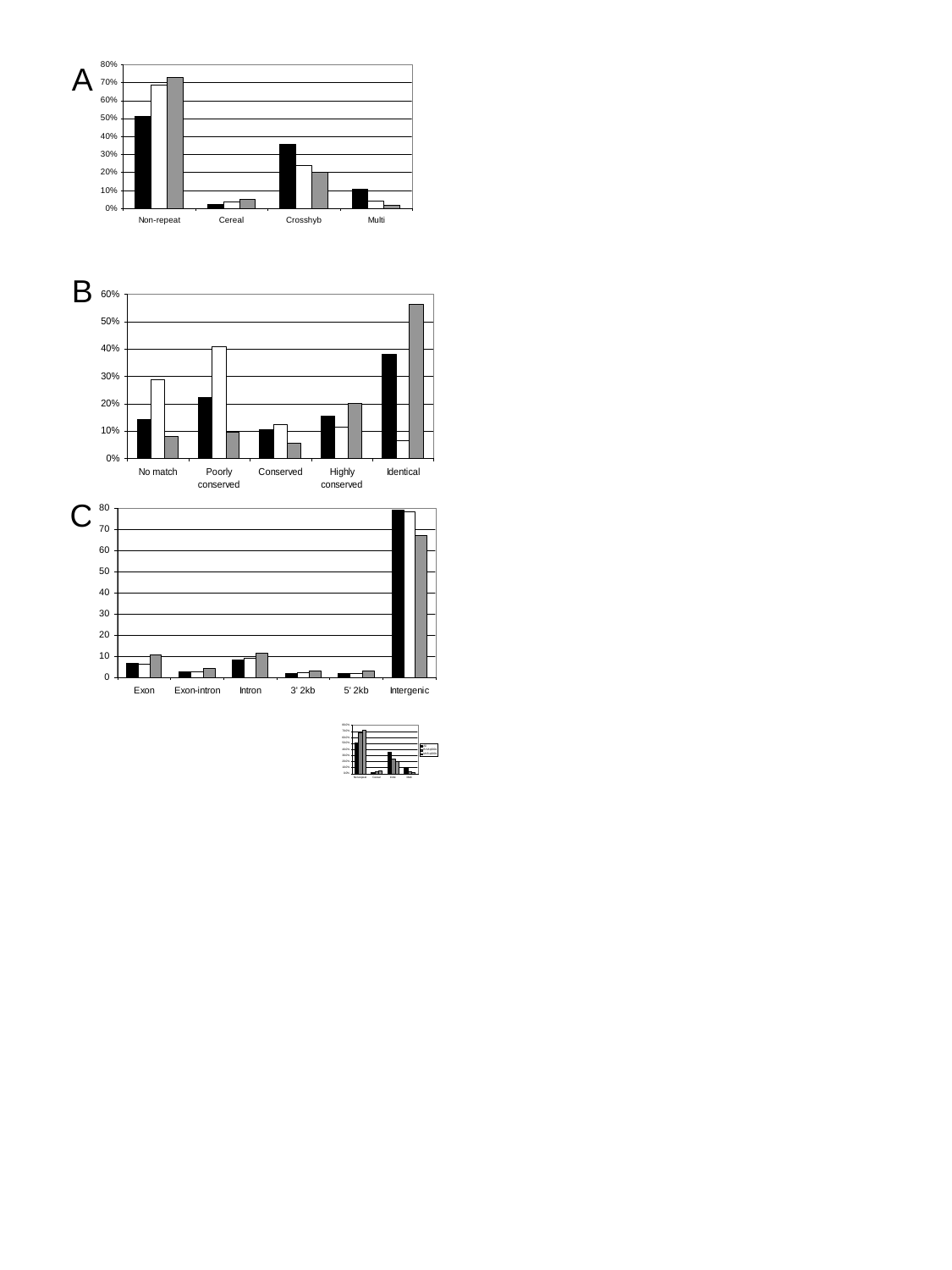

A
B
C

Supplement: Figure S8 — Annotation of probes that exhibit significant (q<0.0001) variation in hybridization to B73 and Mo17 genomic DNA. (A) The percentage of all probes, B73>Mo17 probes and Mo17>B73 probes that are classified as non-repeat, multi-copy, icicle or cereal repeats. (B) For the same sets of probes, the conservation of probe sequence in Mo17 was assessed. (C) The location of significant probes relative to the MGSC working set of genes was also assessed. Each probe was classified as exon, other genic (including intron, UTR, or junctions), or non-genic. (0.10 MB PPT) [file pgen.1000734.s008.ppt]

## Slide 1
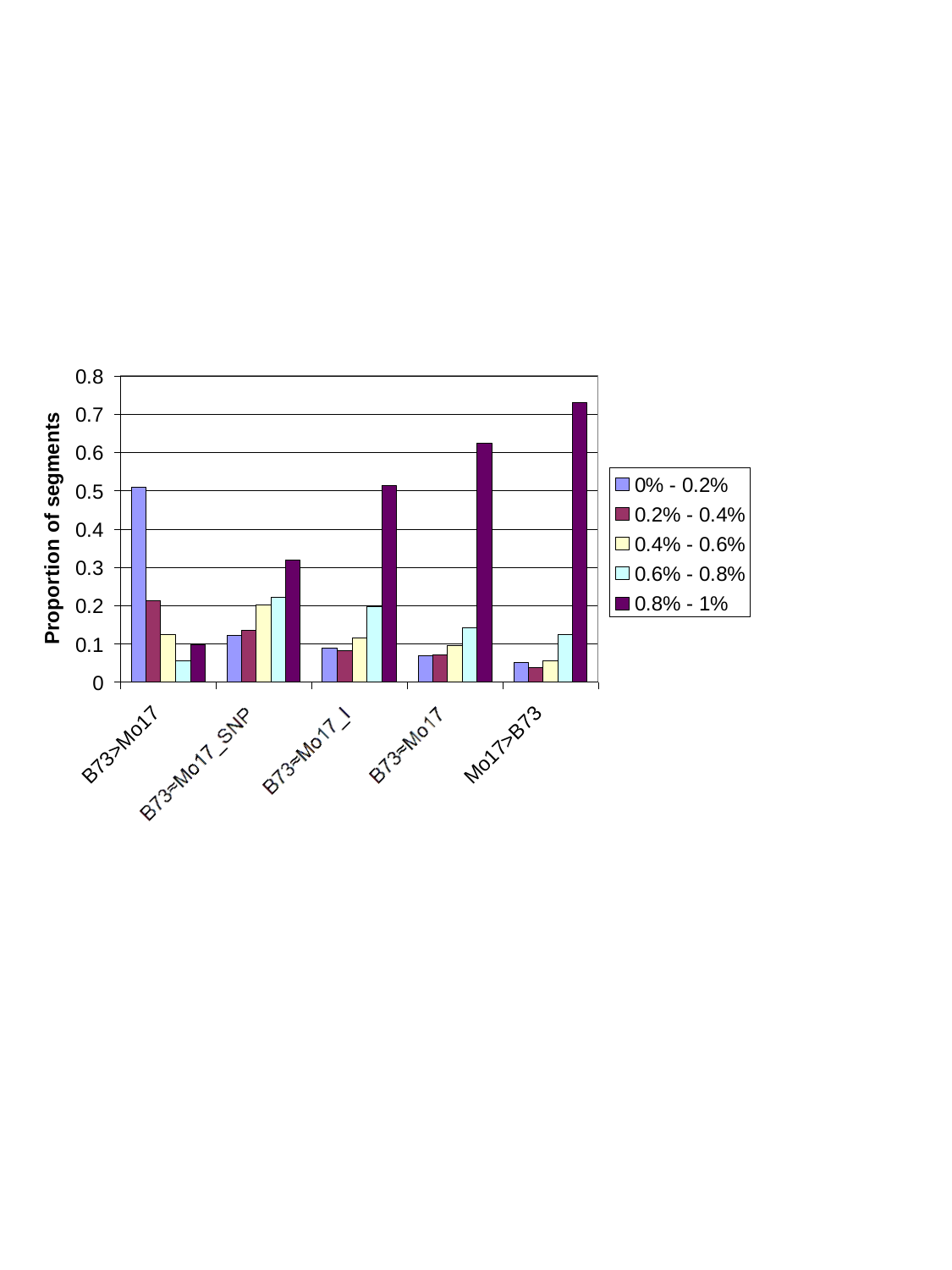

Supplement: Figure S13 — Distribution of Mo17 coverage for DNA segments in each category. The proportion of stringent segments with the specified coverage by the Mo17 454 WGS reads are specified for each category. Note that the coverage statistics are the proportion of non-repetitive bases within the DNA sequence that are covered by Mo17 WGS sequence. (4.44 MB PPT) [file pgen.1000734.s013.ppt]
