# Supplementary material for: Maize Inbreds Exhibit High Levels of Copy Number Variation (CNV) and Presence/Absence Variation (PAV) in Genome Content
Source: PLoS Genet. 2009 Nov 20;5(11):e1000734. doi: 10.1371/journal.pgen.1000734 (PMC2780416; doi:10.1371/journal.pgen.1000734)
Supplement: Figure S2 — Significant hybridization differences are due to structural variation. The B73 and Mo17 sequences for two portions (A and B) of the bz1 locus (sequenced and annotated by Fu and Dooner 2002 and Brunner et al., 2005) were aligned using Vista (Frazer et al., 2004) which displays the percent identity as a sliding window of 100 bp (y-axis is 50% to 100% identity). The location of genes (indicated by light blue sequences in the alignment) and repeat elements (the color-coded track right above the alignments; retrotransposons are shaded pink and transposons are shaded orange) are shown above the VISTA alignment. The log2(Mo17 signal/B73 signal) is shown for each probe in this region. The red probes exhibit significantly different (q<0.0001) signal in B73 and Mo17. The repetitive annotation is shown as a track below the log signal (blue are repetitive probes and black are non-repetitive probes). The blue line indicates a segment with altered hybridization that was identified using DNAcopy. Note that these annotations are based on the genome-wide analysis, not detailed analyses of these regions. The last four probes in (A) and the fist four probes in (B) occur in regions where Mo17 does not have similar sequence at the allelic position but do not show significant differences in hybridization. This suggests that there are examples of sequences that are present in Mo17 but at a non-allelic position. (0.45 MB PPT) [file pgen.1000734.s002.ppt]

## Slide 1
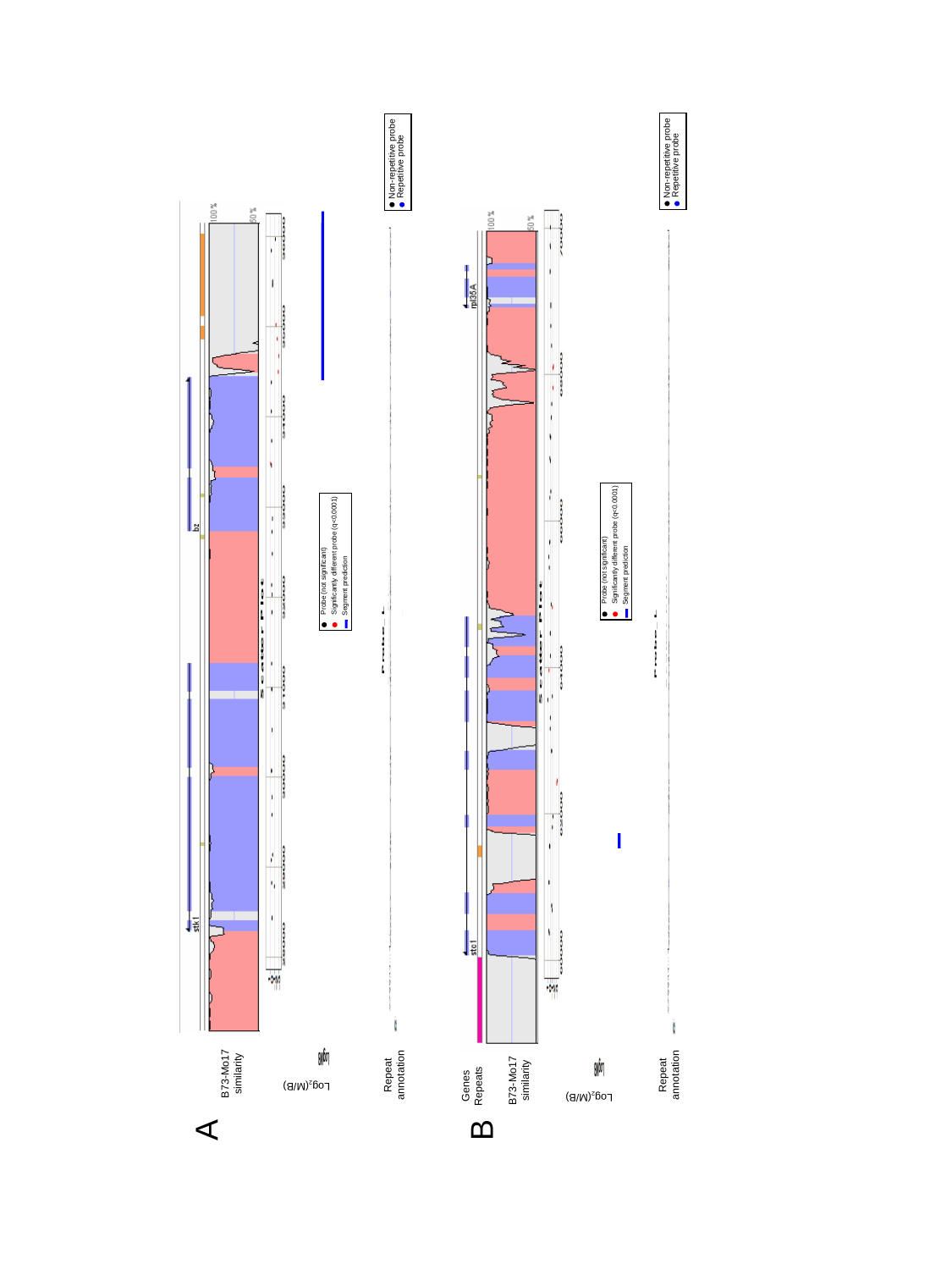

Non-repetitive probe
Non-repetitive probe
Repetitive probe
Repetitive probe
Significantly different probe (q<0.0001)
Probe (not significant)
Segment prediction
Significantly different probe (q<0.0001)
Probe (not significant)
Segment prediction
B73-Mo17 similarity
Repeat annotation
Repeat annotation
B73-Mo17 similarity
Genes
Repeats
Log2(M/B)
Log2(M/B)
A
B
