# Supplementary material for: Maize Inbreds Exhibit High Levels of Copy Number Variation (CNV) and Presence/Absence Variation (PAV) in Genome Content
Source: PLoS Genet. 2009 Nov 20;5(11):e1000734. doi: 10.1371/journal.pgen.1000734 (PMC2780416; doi:10.1371/journal.pgen.1000734)
Supplement: Figure S6 — Volcano and MA plots for classes of repetitive probes. (A) The multi-copy repeat probes (at least 5 copies of >97% identity and coverage) are shown in blue. Many of these probes have high hybridization signals. (B) The crosshyb repeat probes (at least five genomic loci with 90% identity and coverage) are shown in red. These probes rarely show significant differences and have a range of different hybridization values. (C) The cereal repeat probes (similar to sequences in ISU cereal repeat database) are shown in yellow. (0.38 MB PPT) [file pgen.1000734.s006.ppt]

## Slide 1
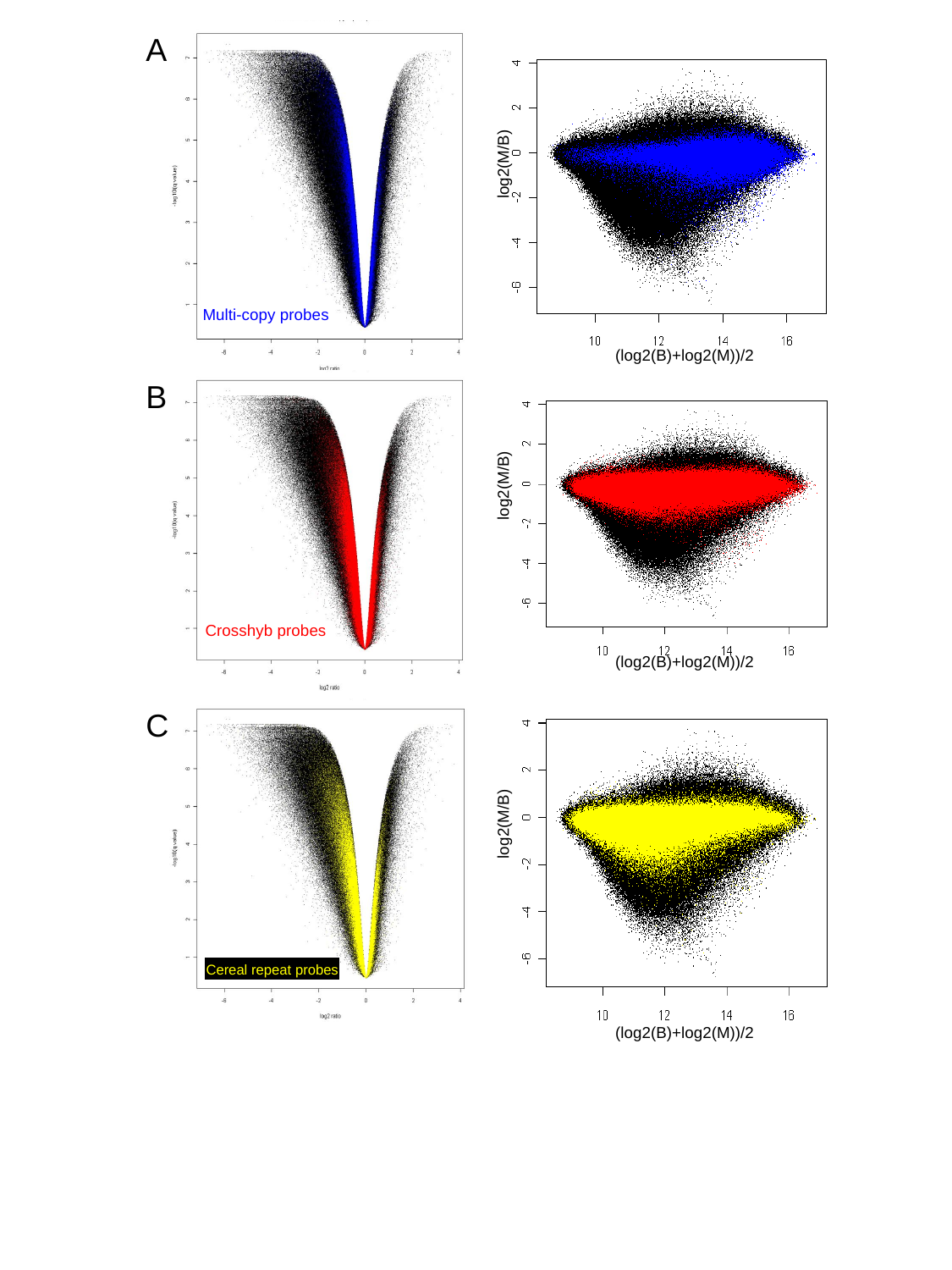

A
log2(M/B)
Multi-copy probes
(log2(B)+log2(M))/2
B
log2(M/B)
Crosshyb probes
(log2(B)+log2(M))/2
C
log2(M/B)
Cereal repeat probes
(log2(B)+log2(M))/2
