# Supplementary material for: Maize Inbreds Exhibit High Levels of Copy Number Variation (CNV) and Presence/Absence Variation (PAV) in Genome Content
Source: PLoS Genet. 2009 Nov 20;5(11):e1000734. doi: 10.1371/journal.pgen.1000734 (PMC2780416; doi:10.1371/journal.pgen.1000734)
Supplement: Figure S7 — Repetitive probes rarely report variation in B73 and Mo17. The chromosomal distribution (x-axis) is shown for each class of repetitive probe relative to the log2(Mo17/B73) (y-axis). (A) The multi-copy repeat probes (at least 5 copies of >97% identity and coverage) are shown in blue and all other probes are shown in gray. (B) The crosshyb repeat probes (at least copies that have 90% identity and coverage) are shown in red and all other probes are shown in gray. (C) The cereal repeat probes (similar to sequences in ISU cereal repeat database) are shown in yellow and all other probes are shown in gray. (0.54 MB PPT) [file pgen.1000734.s007.ppt]

## Slide 1
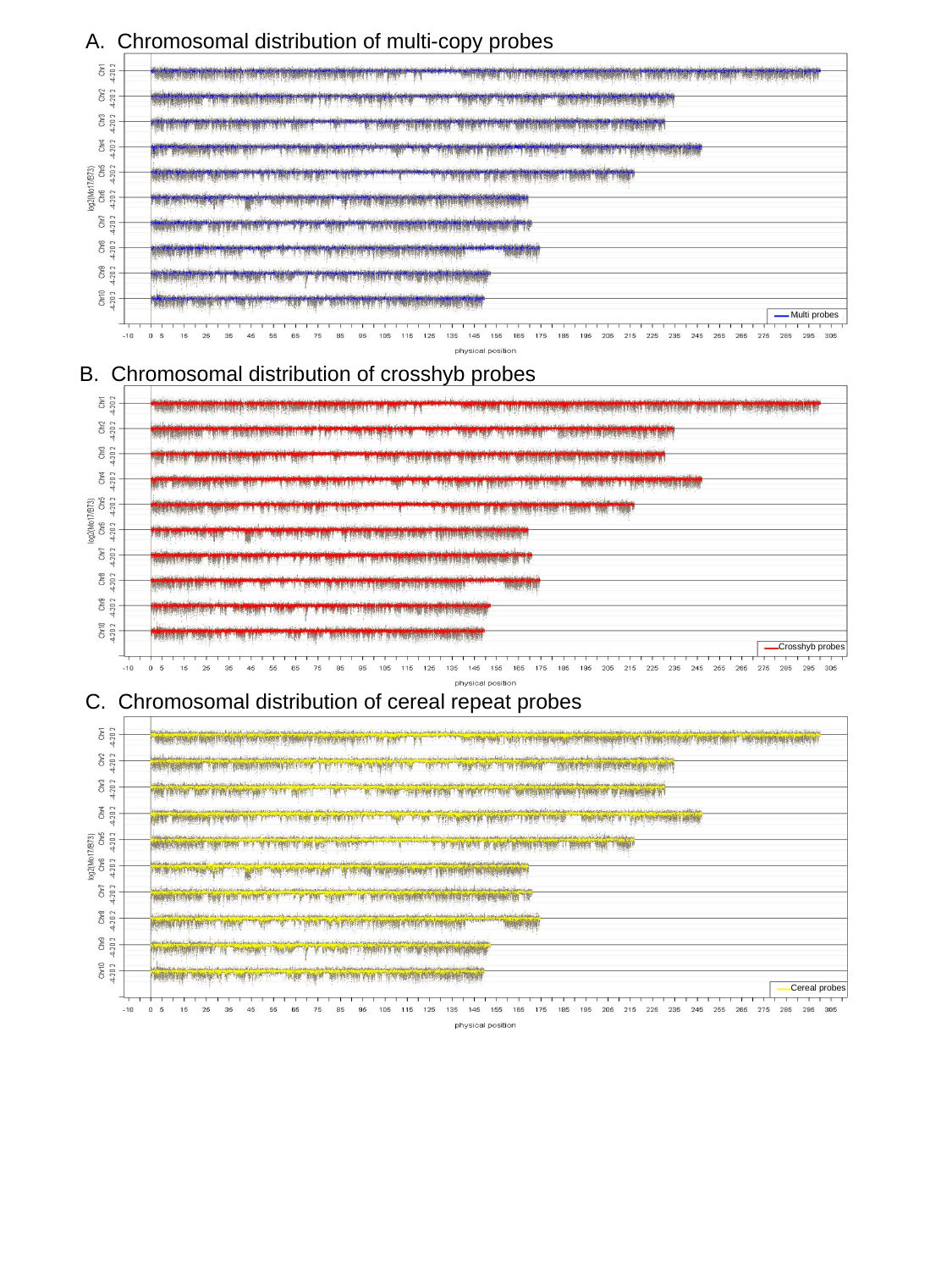

A. Chromosomal distribution of multi-copy probes
Multi probes
B. Chromosomal distribution of crosshyb probes
Crosshyb probes
C. Chromosomal distribution of cereal repeat probes
Cereal probes
