# Supplementary material for: Maize Inbreds Exhibit High Levels of Copy Number Variation (CNV) and Presence/Absence Variation (PAV) in Genome Content
Source: PLoS Genet. 2009 Nov 20;5(11):e1000734. doi: 10.1371/journal.pgen.1000734 (PMC2780416; doi:10.1371/journal.pgen.1000734)
Supplement: Figure S9 — Volcano and MA plots for differing levels of conservation in Mo17 sequence. Each probe was compared to the Mo17 454 WGS sequence (provided by the Joint Genome Institute) and classified as perfect match (100% identity and coverage), highly conserved (>97% identity and coverage), conserved (>90% identity and coverage), poorly conserved (>75% identity and 70% coverage) or no match. The distribution of signals and variation for each type of probe are shown using volcano plots and MA plots. The pie chart shows the relative proportion of each type of probe. (0.50 MB PPT) [file pgen.1000734.s009.ppt]

## Slide 1
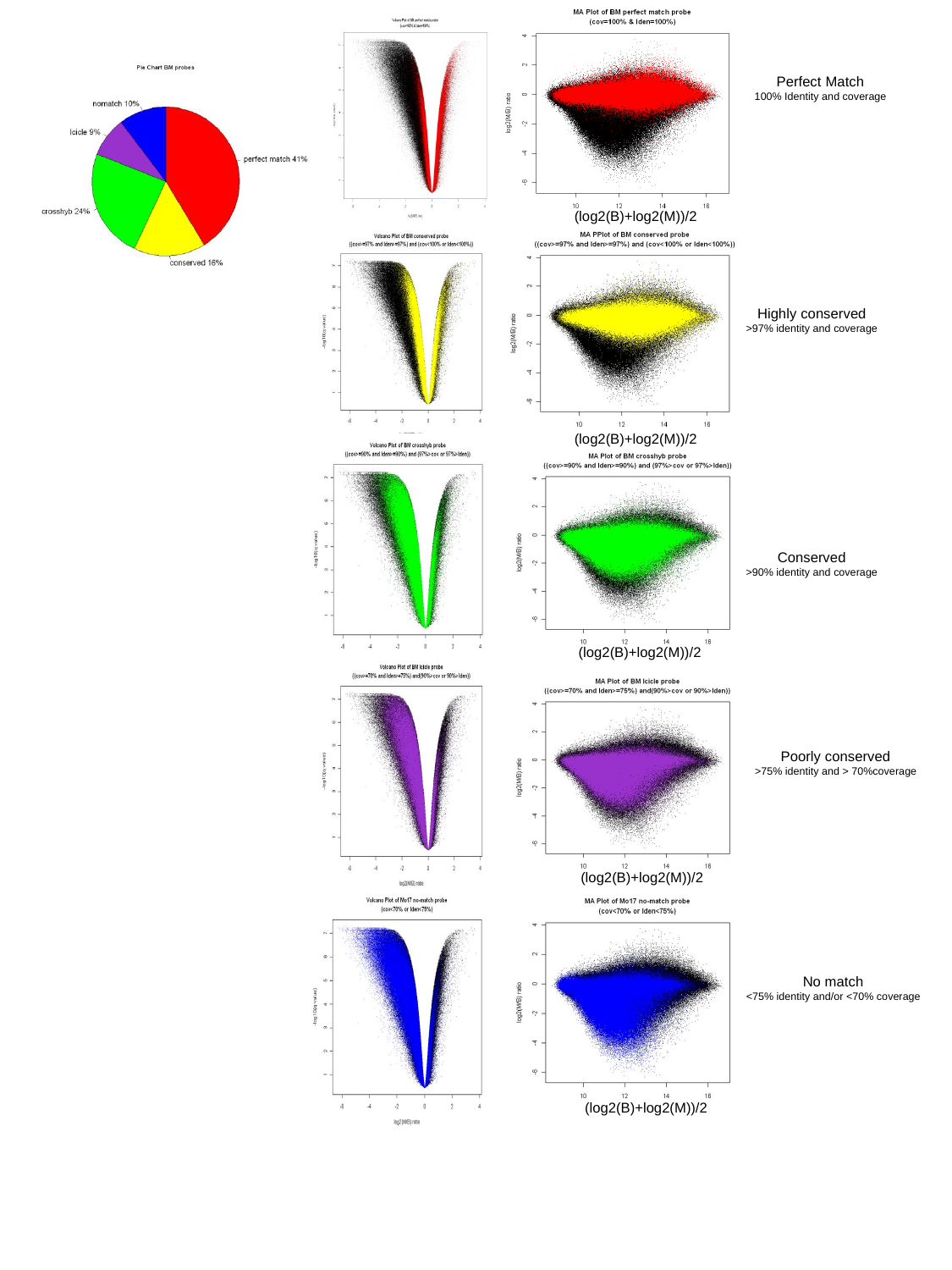

Perfect Match
100% Identity and coverage
(log2(B)+log2(M))/2
Highly conserved
>97% identity and coverage
(log2(B)+log2(M))/2
Conserved
>90% identity and coverage
(log2(B)+log2(M))/2
Poorly conserved
>75% identity and > 70%coverage
(log2(B)+log2(M))/2
No match
<75% identity and/or <70% coverage
(log2(B)+log2(M))/2
