# Supplementary material for: Maize Inbreds Exhibit High Levels of Copy Number Variation (CNV) and Presence/Absence Variation (PAV) in Genome Content
Source: PLoS Genet. 2009 Nov 20;5(11):e1000734. doi: 10.1371/journal.pgen.1000734 (PMC2780416; doi:10.1371/journal.pgen.1000734)
Supplement: Figure S10 — Rates of variation and chromosomal distribution of probes with different levels of B73-Mo17 sequence conservation. The boxes indicate the positions of the centromeres (from Wolfgruber et al.[72]). (0.18 MB PPT) [file pgen.1000734.s010.ppt]

## Slide 1
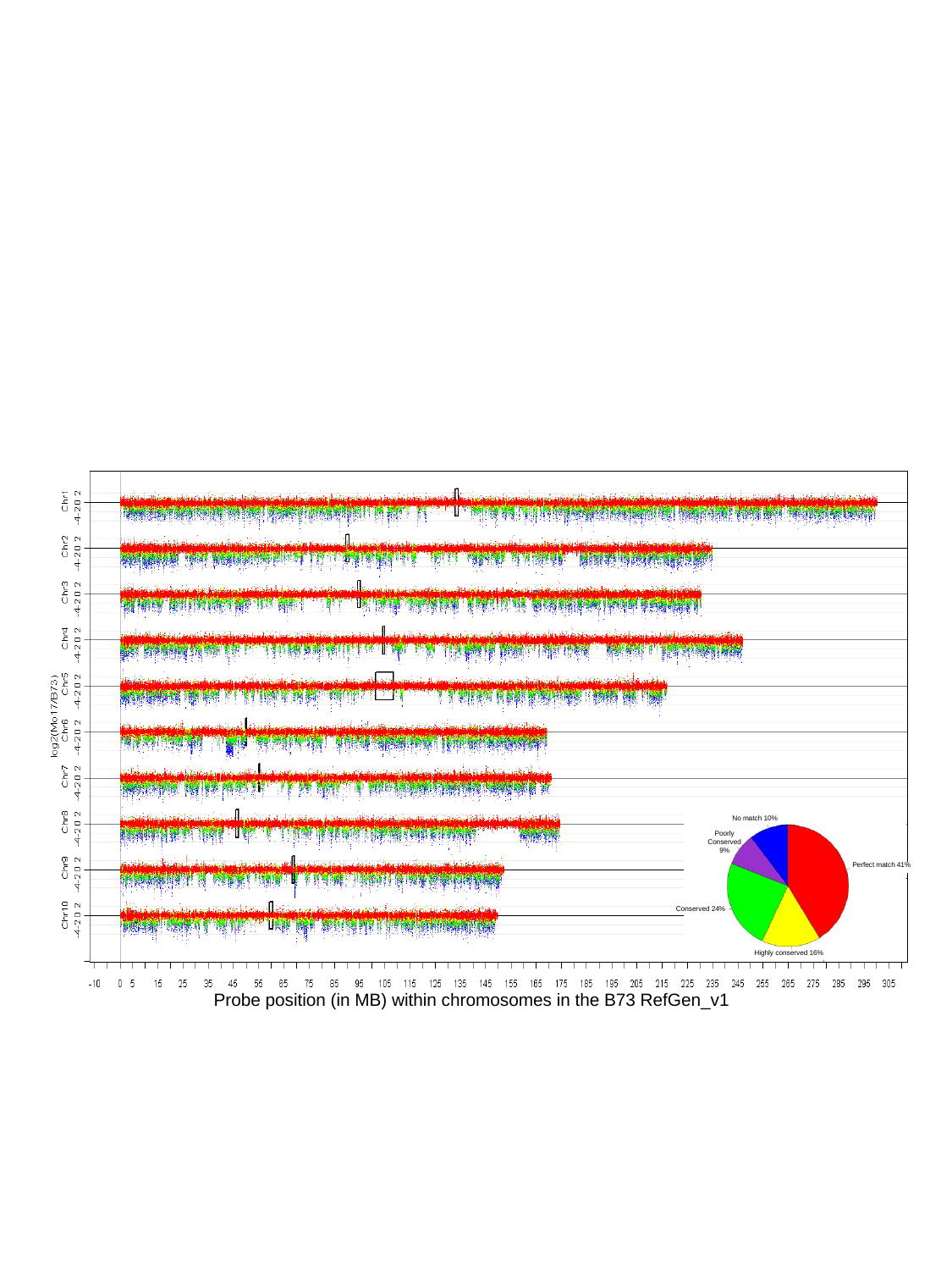

No match 10%
Poorly
Conserved
9%
Perfect match 41%
Perfect match
Highly conserved
Conserved 24%
Conserved
Poorly conserved
No match
Highly conserved 16%
Probe position (in MB) within chromosomes in the B73 RefGen_v1
