# Supplementary material for: Maize Inbreds Exhibit High Levels of Copy Number Variation (CNV) and Presence/Absence Variation (PAV) in Genome Content
Source: PLoS Genet. 2009 Nov 20;5(11):e1000734. doi: 10.1371/journal.pgen.1000734 (PMC2780416; doi:10.1371/journal.pgen.1000734)
Supplement: Figure S11 — Genomic regions of low (A) or high (B) levels of structural variation. The log2(Mo17/B73) hybridization intensities are plotted for a region on chromosome 8 (A) with low levels of probes that detect structural variation. In (B) the hybridization intensities are plotted for all of the probes within a region on chromosome 6 with high levels of probes that detect structural variation. (0.15 MB PPT) [file pgen.1000734.s011.ppt]

## Slide 1
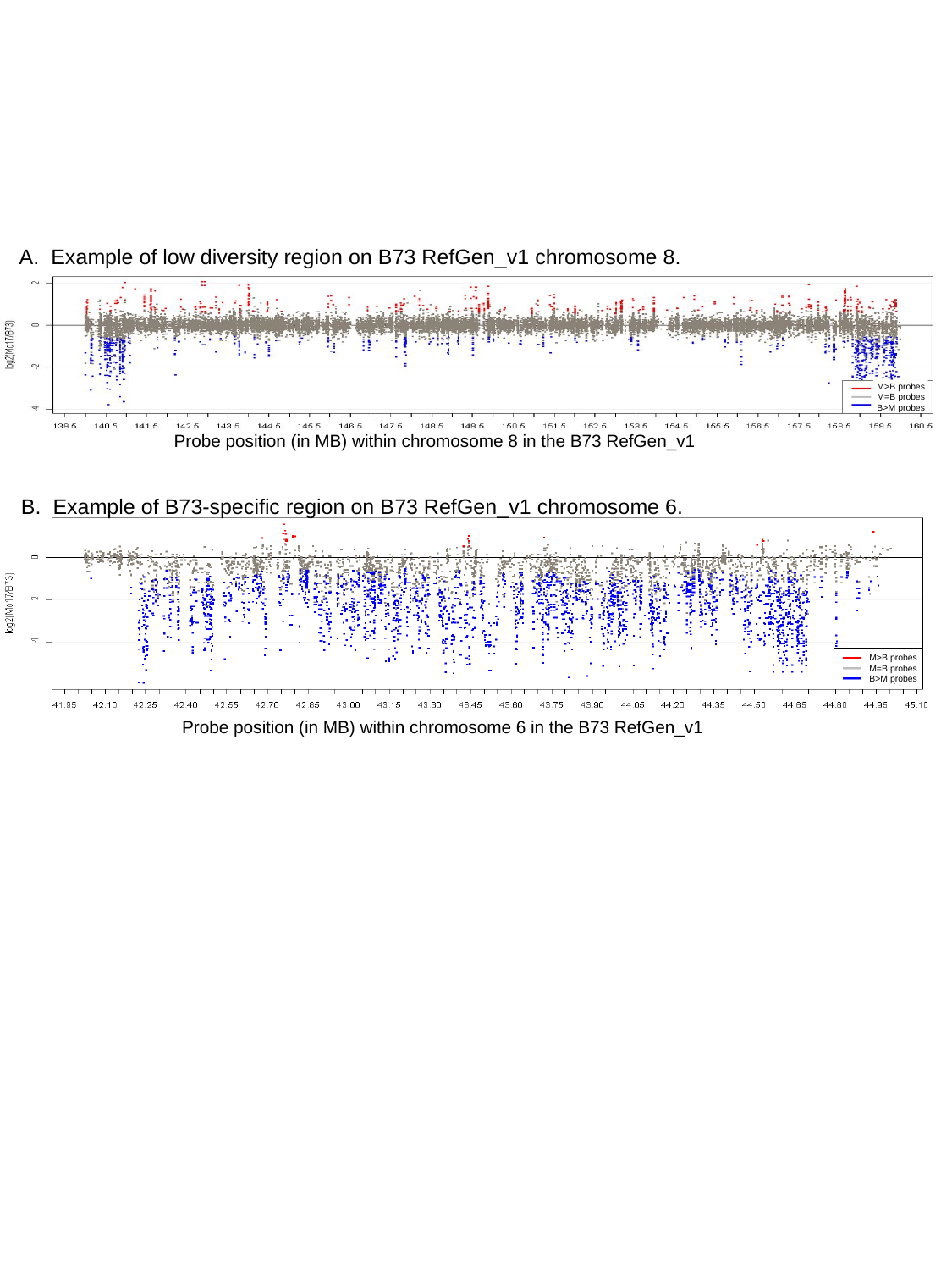

A. Example of low diversity region on B73 RefGen_v1 chromosome 8.
M>B probes
M=B probes
B>M probes
Probe position (in MB) within chromosome 8 in the B73 RefGen_v1
B. Example of B73-specific region on B73 RefGen_v1 chromosome 6.
M>B probes
M=B probes
B>M probes
Probe position (in MB) within chromosome 6 in the B73 RefGen_v1
