# Supplementary material for: Maize Inbreds Exhibit High Levels of Copy Number Variation (CNV) and Presence/Absence Variation (PAV) in Genome Content
Source: PLoS Genet. 2009 Nov 20;5(11):e1000734. doi: 10.1371/journal.pgen.1000734 (PMC2780416; doi:10.1371/journal.pgen.1000734)
Supplement: Figure S12 — Annotation of probes that are within stringent segments that are present only in B73, or are higher in copy number in B73 or in Mo17. (A) The proportion of probes within stringent segments that are classified as non-repeat, multi-copy, icicle or cereal repeats. (B) For the same sets of probes, the conservation of probe sequence in Mo17 was assessed. (C) The location of probes relative to genes was also assessed. Each probe was classified as exon, exon-intron, intron, 5′ 2000bp or 3′ 2000bp. (0.19 MB PPT) [file pgen.1000734.s012.ppt]

## Slide 1
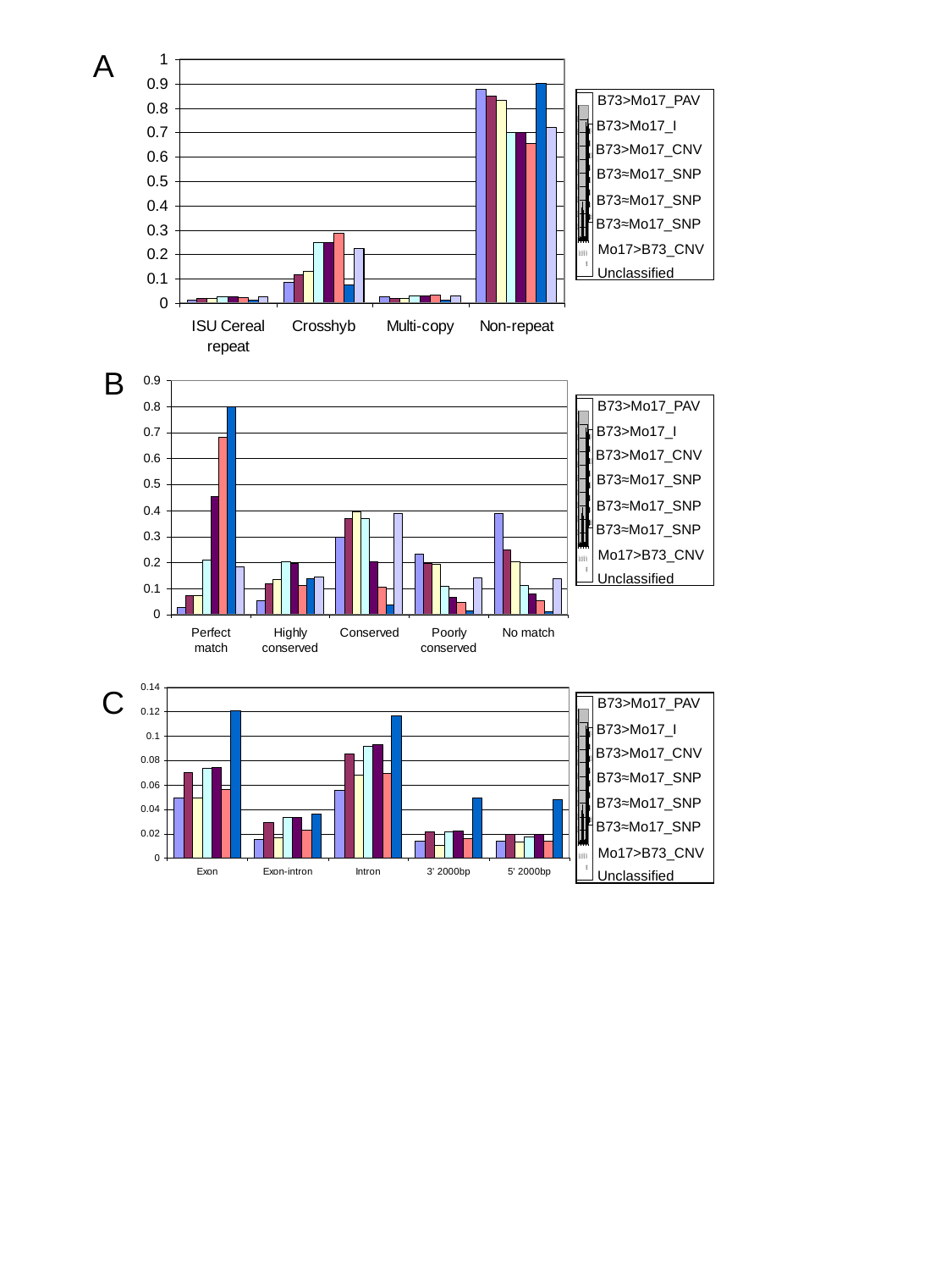

A
B73>Mo17_PAV
B73>Mo17_I
B73>Mo17_CNV
B73≈Mo17_SNP
B73≈Mo17_SNP
B73≈Mo17_SNP
Mo17>B73_CNV
Unclassified
B
B73>Mo17_PAV
B73>Mo17_I
B73>Mo17_CNV
B73≈Mo17_SNP
B73≈Mo17_SNP
B73≈Mo17_SNP
Mo17>B73_CNV
Unclassified
C
B73>Mo17_PAV
B73>Mo17_I
B73>Mo17_CNV
B73≈Mo17_SNP
B73≈Mo17_SNP
B73≈Mo17_SNP
Mo17>B73_CNV
Unclassified
